# National Initiatives on Salt Substitutes: Scoping Review

**DOI:** 10.2196/45266

**Published:** 2023-11-17

**Authors:** Bingxuan Kong, Shanyue Yang, Jiewei Long, Yuhan Tang, Yang Liu, Zeng Ge, Shuang Rong, Yangfeng Wu, Gangqiang Ding, Yuexin Yang, Ping Yao, Chao Gao

**Affiliations:** 1 National Institute for Nutrition and Health, Chinese Center for Disease Control and Prevention Beijing China; 2 Department of Nutrition and Food Hygiene, School of Public Health, Tongji Medical College, Huazhong University of Science and Technology Wuhan China; 3 Cardiovascular Health Program, Vital Strategies (USA) Jinan Representative Office Jinan China; 4 Department of Nutrition, School of Public Health, Wuhan University Wuhan China; 5 Peking University Clinical Research Institute, Peking University Beijing China; 6 Key Laboratory of Trace Element Nutrition of National Health Commission Beijing China; 7 Chinese Nutrition Society Beijing China; 8 Ministry of Education Key Laboratory of Environment, School of Public Health, Tongji Medical College, Huazhong University of Science and Technology Wuhan China

**Keywords:** salt substitute, nutrition policy, initiatives, strategies

## Abstract

**Background:**

Salt substitutes are edible salts that contain less sodium chloride owing to its partial substitution with other minerals, which serve as an important and effective intervention and public health strategy targeting hypertension and its secondary diseases, despite a small degree of controversy.

**Objective:**

This study aimed to identify the current salt substitute initiatives in various nations and intergovernmental organizations (IGOs) worldwide and summarize their types and characteristics.

**Methods:**

A scoping review was performed based on Arksey and O’Malley’s framework and the latest guidance from the Joanna Briggs Institute. Searches were conducted on Google; government websites on food, health, and other related topics; PubMed; Web of Science; and Google Scholar from January 2022 to May 2022. Initiatives related to salt substitutes that were included in the study focused on the involvement of governments or IGOs through the publication of standards, actions, collaborations, funding, and so on. Data were extracted into Microsoft Excel (version 2019; Microsoft Corp) based on predefined items and analyzed using narrative synthesis and frequency count methods.

**Results:**

A total of 35 initiatives from 11 countries (of which 9 are high-income countries) and 3 IGOs were identified. We classified all salt substitute initiatives into 5 types, namely benefit-risk assessments and cautions; plans and actions; regulations and standards; labels; and food reformulation, cooperation with the food industry, and media. More than half (18/35, 51%) of the salt substitute initiatives were launched within the past 5 years. Except for regulations and standards, salt substitute initiatives are, in general, part of the salt reduction framework. No nation or IGO has yet reported on the monitoring and implications of the use of salt substitutes.

**Conclusions:**

Despite the limited number of salt substitute initiatives worldwide at present, a review on the different types and characteristics of such initiatives could be helpful in providing a reference for policy makers and stakeholders. Given the great potential of salt substitutes in improving hypertension and stroke, we call on more nations to pay attention to these substitutes and propose salt substitute initiatives in line with their national conditions.

## Introduction

### Background

Hypertension is a common chronic disease, accounting for approximately 11 million deaths, or approximately one-fifth of all deaths, in 2019 [1]. Hypertension and cardiovascular disease continue to be the leading cause of disability and death among patients with COVID-19 during the pandemic, who are affected by the lack of health care resources caused by the spread of the pandemic [2,3].

Salt reduction is one of the most important public health strategies for the prevention and control of hypertension. In China, a high-sodium diet was identified as the third most significant risk factor for both the number of deaths and the percentage of disability-adjusted life-years in 2017, accounting for over 1.5 million fatalities [4]. The World Health Organization (WHO) advocated for a 30% decrease in population salt intake as one of the primary interventions to reduce premature death from noncommunicable diseases [5]. Although the WHO recommends that adults consume <5 g of salt or 2000 mg of sodium per day, most people consume up to twice the recommended amount: an average of 9 to 12 g of salt per day [6]. A systematic review of global salt reduction initiatives shows that as of 2019, a total of 96 countries or regions have adopted different structural or regulatory approaches to salt reduction [7]. At the same time, the WHO also recommended that adults consume at least 3510 mg (90 mmol) of potassium per day [8]. Potassium has a blood pressure control effect [9], and potassium intake has been shown in many randomized controlled trials and modeling studies to be a good initiative for blood pressure and cardiovascular disease control [10,11]. In addition, sodium-potassium interaction is an important mechanism of hypertension [12]. However, the amount of potassium intake is generally not enough, such as the 1997 mg/day reported by the United States [13]. It was also reported that potassium intake and 24-hour urinary potassium excretion were less than half of the current recommendations in China [14,15].

As an emerging approach to salt reduction, salt substitutes are gaining attention in more and more countries. Salt substitutes or low-sodium salts usually refer to table or cooking salts that either do not contain sodium chloride or contain lower levels of sodium chloride owing to its partial replacement with potassium chloride, magnesium sulfate, or other minerals [16]. Salt substitutes have a similar taste to that of regular table salt. With an extra amount of potassium added, it serves as an ideal solution to reduce sodium and increase potassium and other mineral intake at the same time. Studies have shown the benefits of salt substitutes in controlling blood pressure and reducing the risk of cardiovascular disease and mortality [17]. A global environmental scan of salt substitution products showed that as of September 2020, a total of 87 salt substitutes were available in 47 countries worldwide [16]. However, many people still have concerns about the risk of adverse reactions associated with the intake of salt substitutes, such as hyperkalemia [18].

### This Review

Salt reduction is one of the most important methods of preventing and controlling noncommunicable diseases in most countries [7]. Among others, salt substitute, as a new means of salt reduction, is gradually gaining attention and being popularized. However, there is no research summarizing and analyzing national salt substitute initiatives. In this context, the objectives of this scoping review were to (1) search for and document existing national salt substitute initiatives (before May 2022), (2) analyze the types and characteristics of national salt substitute initiatives, and (3) provide recommendations on the promotion and regulation of salt substitute applications in different regions and countries.

## Methods

### Approach

The scoping review was developed based on Arksey and O’Malley’s [19] framework and the latest guidance from the Joanna Briggs Institute [20]. We reported according to the PRISMA-ScR (Preferred Reporting Items for Systematic Reviews and Meta-Analyses Extension for Scoping Reviews) recommendations [21]. The PRISMA-ScR checklist is presented in Multimedia Appendix 1 [21].

### Definition of Salt Substitutes and National Initiatives on Salt Substitutes

The expression of salt substitutes and the content of sodium varied across countries and regions for different products. Salt substitutes and low-sodium salts were considered synonymous in this study and defined as table salts or cooking salts whose sodium chloride was replaced with other minerals such as potassium chloride or magnesium sulfate, including sodium-reduced and sodium-free salts.

National initiatives on salt substitutes referred to initiatives related to salt substitutes that national governments and subsidiary organizations were engaged in through cooperation, development, publication, and implementation, as described in the *Eligibility Criteria* section. Nations in this study refer to the 193 United Nations (UN) member states [22]. Notably, intergovernmental organizations (IGOs) were organizations with international legal personalities established through treaty or other agreement between member states [23,24]. The main IGOs searched included UN agencies and regional organizations related to health, food, and agriculture [25] (all nations and IGOs searched in this study are listed in Multimedia Appendix 2).

### Eligibility Criteria

The inclusion criteria were as follows: (1) participation of the national government (through funding, commissioning, the publication of standards, etc); (2) a national document or statement stressing on salt reduction and including the use of salt substitutes as a strategy; (3) a salt substitute program developed by the government in partnership with the food or salt industries; (4) a scheme about the labels, slogans, ingredient lists, etc printed on the package of salt substitute or the dietetic product with salt substitutes; and (5) mainstream media initiatives, consumer education, and other means used to increase knowledge on, improve attitudes toward, and behaviors related to salt substitutes.

The exclusion criteria were as follows: (1) salt substitute initiatives without the participation of nations or IGOs, for instance, initiatives led by nongovernmental organizations or local governments, and (2) the definition of salt substitutes differs from that used in this study, for example, flavor enhancers such as pepper and lemon.

### Search Strategy

Three methods were used to conduct the research: government website search, Google search, and literature search. The search concepts included salt substitutes, countries, IGOs, portal function, government portals, states, and initiatives. Multimedia Appendix 2 lists the specific search process, all the keywords used to address the different aspects of the search concepts, and all the countries and IGOs involved. The government websites were obtained by searching the name of the 193 UN member states and the keywords related to government portals on Google Chrome with Boolean operators. First, keywords related to concepts such as government portals and portal function and country names were used to sort out government portal websites on health, food, medicine, and other related topics on Google Chrome, followed by a more in-depth exploration of keywords related to salt substitutes on these websites. With regard to IGOs, the official websites of a total of 66 IGOs related to global or regional political, economic, health, and agricultural cooperation were searched on Google Chrome. In addition, Google Chrome was directly used for keywords related to the concepts of salt substitutes, initiatives, nations, and IGOs with suitable Boolean operators. The first 5 pages of each Google search result were evaluated for eligibility, which usually contain almost all the information needed. All UN member countries were searched, with no language limitations set. If a country’s official language was not English, the built-in translation service in Google Chrome was used to translate this language into English [26]. Finally, literature searches on PubMed, Web of Science, and Google Scholar were carried out using the corresponding search keywords and search formulas shown in Multimedia Appendix 2. The language of the literature search was restricted to English. All information was collected between January 2022 and May 2022.

### Data Selection and Extraction

After receiving training, BK, SY, and JL individually conducted data identification and retrieval based on the established search strategy and eligibility criteria under the supervision of CG. The collected data was subsequently summarized. The checks during the summarization phase primarily focused on identifying and removing duplicates. Discrepancies were reviewed independently by CG, YT, YL, and PY and resolved through discussion. Data accuracy was independently checked by CG, PY, ZG, SR, YW, GD, and YY. The data eventually included in this study were extracted by BK and YL into a standardized table in a Microsoft Excel (version 2019; Microsoft Corp) sheet, which was independently reviewed by CG and PY and discussed in case of discrepancies. For government documents, the key information extracted included the (1) name of the document, (2) initiative enactment time, (3) the nation which published the document, (4) ministry that mandated the document, and (5) key content. From academic literature, we collected the following information: (1) the type of literature (article, review, letter, etc), (2) the year of publication, (3) the methods used, (4) the study population, and (5) relevant results.

### Data Analysis

Data analysis was mainly performed through narrative synthesis and frequency count [27]. The research team collated and categorized the information obtained, summarizing and analyzing them through words and text. Data were also categorized based on the date of the publication of the initiatives, the WHO regional categorization, and the countries’ income levels in the 2022 World Bank classification. Software used included Microsoft Excel 2019, ArcMap (version 10.8; Environment Systems Research Institute), and GraphPad Prism (version 8.0.1; Graph Pad Software, Inc).

### Ethical Considerations

This study did not involve human participants, human participant research ethics, or related secondary analyses; therefore, ethics approval and consent to participate were not required.

## Results

### Search Results

**Figure 1:**
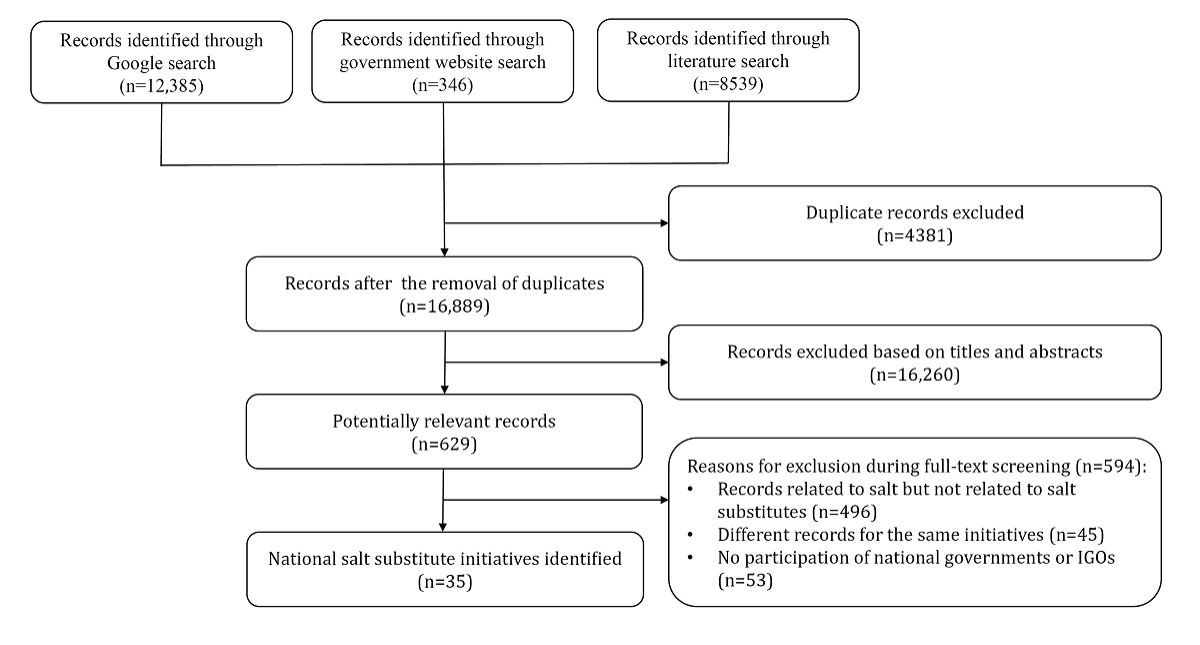
Flow diagram of salt substitute initiatives identification. IGO: intergovernmental organization.

A total of 16,889 records were identified after removing duplicates. On the basis of titles and abstracts, 16,260 (92.28%) records were excluded. After full-text reading, of the remaining 629 records, 594 (94.4%) were excluded for the reasons listed in Figure 1, the PRISMA-ScR flow diagram. As a result, 35 initiatives from 11 countries and 3 IGOs were eligible. Multimedia Appendix 3 [28-63] contains all the national and IGO initiatives for salt substitutes that were included.

**Figure 2:**
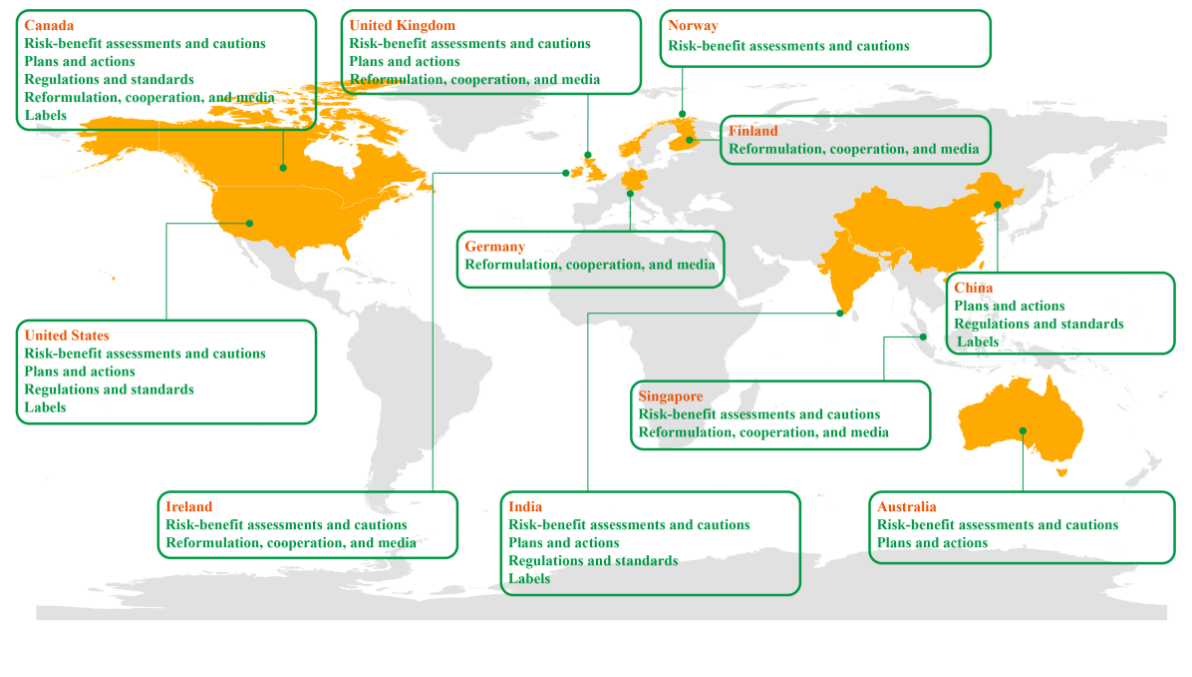
National salt substitute initiatives.

The 11 countries were the United Kingdom, Germany, Norway, Ireland, and Finland in Europe; the United States and Canada in North America; Singapore, China, and Australia in East Asia and the Pacific; and India in South Asia (Figure 2). Except for China, which is an upper-middle-income country, and India, which is a lower-middle-income country according to the recently updated (2022) World Bank income ranking, all countries are high-income countries [64]. The 3 IGOs are the European Union (EU), the Eurasian Economic Union (EAEU), and The Codex Alimentarius Commission (CAC). Salt substitute initiatives were divided into five types, namely (1) benefit-risk assessments and cautions; (2) plans and actions; (3) regulations and standards; (4) labels; and (5) food reformulation, cooperation with the food industry, and media, all of which are detailed in Multimedia Appendix 4.

The total number of countries classified as belonging to the given regions according to the WHO regional categorization is shown within parentheses. Some initiatives fall under several categories.

### Types and Characteristics of National Salt Substitute Initiatives

#### Benefit-Risk Assessments and Cautions

The benefit-risk assessments of salt substitutes are usually carried out by a nutrition-related scientific committee appointed by the relevant government food safety department, providing recommendations on the use of salt substitutes. The benefit-risk assessments are an important part of determining national attitudes toward salt substitutes, which are a prerequisite for some countries to develop salt reduction–related initiatives. Assessments have been conducted in the United Kingdom [28], Ireland [29,30], Norway [31,32], and Australia [33].

In the United Kingdom, the Scientific Advisory Committee on Nutrition and the Committee on Toxicity of Chemicals in Food, Consumer Products and the Environment published *Potassium-Based Sodium Replacers: Assessment of the Health Benefits and Risks of Using Potassium-Based Sodium Replacers in Foods in the UK*, which was an important evaluation of evidence for the application and promotion of salt substitutes [28]. The Scientific Advisory Committee on Nutrition and Committee on Toxicity of Chemicals in Food, Consumer Products and the Environment applied the benefit-risk analysis for foods methodology to hypothesize the benefits and risks of reduced sodium intake and increased potassium intake in the general UK population by replacing 15% to 25% of sodium with potassium (potassium chloride, potassium carbonate, or potassium bicarbonate). This report recognized that the potential benefits of salt substitutes outweighed the potential risks and that although the impact at the individual level would be small, salt substitutes would have a beneficial effect on a large proportion of the UK population. The report recommended that the government encourage food businesses to use salt substitutes, but the level of potassium in foods and the types of foods in which salt substitutes are used needed to be monitored.

In addition to the United Kingdom, Norway conducted 2 assessments, one in 2014 and another in 2021. In 2014, the Norwegian Scientific Committee for Food and Environment, commissioned by the Norwegian Food Safety Authority and The Norwegian Directorate of Health, conducted a benefit-risk assessment by assuming the benefits and risks of 3 scenarios in which 30%, 50%, or 70% of sodium chloride was replaced by potassium, concluding that it was reasonable to anticipate that the percentage of persons likely to face an increased risk was far greater than the percentage of persons likely to benefit [31]. In 2021, the scientific committee was commissioned again to hypothesize the potential health effects of replacing 0% to 30% of sodium chloride with potassium chloride, and the report has not yet been published [32].

Ireland conducted *Salt and Health: Review of the Scientific Evidence and Recommendations for Public Policy in Ireland* in 2005 and 2016 (revision 1). In 2005, the scientific committee indicated that salt substitutes may increase susceptibility of the health of some populations (including those with type 1 diabetes, chronic renal insufficiency, end-stage renal disease, severe heart failure, or adrenal insufficiency) and did not contribute to lowering the population salt taste thresholds [29]. However, in 2016, the scientific committee concluded that the potassium intake at that time in the Irish diet was not enough and that the use of potassium-based salt replacement ingredients by the food industry could help supplement potassium intake among Irish people. However, the reduction of salt taste thresholds in the population was a major requirement for the food industry. Because the use of salt substitutes was not the preferred way to reduce the thresholds of salt taste, it should only be considered if reducing the sodium content independently could be detrimental to food safety or the physical or organoleptic properties of the food. Meanwhile, the scientific committee recommended that the Irish Food Safety Authority develop guidelines for the food industry regarding the use of potassium and other mineral salt replacements and make these guidelines available to all susceptible groups, that the sodium and potassium content of foods continue to be monitored, and that sodium and potassium intake be reassessed regularly [30].

The Australian Government Department of Health published *Food Reformulation: Risk Assessment* in 2020, which stated that salt substitutes did not necessarily bring about a change in population consumption patterns. In addition, potassium-based replacements could affect the health of susceptible people (“with major impairment of renal function because of chronic kidney disease or other morbidity, and those taking medications such as angiotensin-converting enzyme inhibitors and potassium-sparing diuretics that reduce renal excretion of potassium”). The report recommended that product monitoring and the monitoring of temporal trends in the incidence of hyperkalemia be performed when using salt substitutes and that clear labeling of relevant foods and sound physician recommendations be made for patients at risk [33].

To reduce the potential harm caused by salt substitutes for the higher-risk individuals mentioned earlier, some countries have proposed cautions for salt substitutes, including the recommendation that susceptible groups should use salt substitutes with caution or under professional supervision. For example, in Singapore, salt substitutes are included in the precautions for the use of potassium chloride [34] and the management of chronic heart failure [35]. In China, a warning label (shown in the *Labels* section) is required on the package of salt substitutes [36].

Despite the risk of hyperkalemia that potassium chloride may pose, since 1983, the US Code of Federal Regulations has announced that potassium chloride is generally recognized as safe as a direct human food ingredient when used in accordance with the current good manufacturing practice conditions of use (as a flavor enhancer, flavoring agent, nutrient supplement, pH control agent and stabilizer, or thickener) [37].

#### Plans and Actions

Plans and actions mainly refer to a series of guidelines and solution plans formulated and released by the government for specific problems, such as salt reduction, nutrition, and pollution. The use of salt substitutes in salt reduction plans or actions has been noted in 5 nations (the United Kingdom, Canada, Australia, China, and India).

Guidelines usually address 2 main aspects of salt substitute use. First, the processed food industry should consider reducing the salt content in their products to produce low-salt foods and then consider the use of salt substitutes when necessary (as proposed by the United Kingdom [38] and Canada [39]). Second, when using salt substitutes, attention should be paid to their safety and efficacy. Besides benefit-risk assessments, the disclosure of potassium content on nutrition labels, monitoring of potassium intake, and technological innovations are all possible approaches (as proposed by Canada [39] and Australia [40]). The focus of the salt reduction initiatives in China is different from that of the salt reduction initiatives in other countries, as the Chinese diet is predominantly homemade, especially in rural areas. Because of the high sodium intake, very low potassium intake, and high cardiovascular disease burden in these areas, the initiatives recommended adjusting the salt supply in the whole population while promoting and popularizing low-sodium and potassium-enriched salt. Baseline data collection and follow-up studies were conducted in a nationally representative population using dietary surveys and 24-hour urinary potassium excretion test [41]. In its book *Do You Eat Right*, the Food Safety and Standards Authority of India acknowledged the potential of salt substitutes for salt reduction but also noted that salt substitute brands were currently limited on the market and that salt substitutes should be used only under medical supervision [42].

In addition, although the US *Dietary Guidelines for Americans (2020-2025)* did not address potassium-rich salt substitutes, they stated that Americans’ potassium intake was low and sodium intake should be limited and that the requirements for the nutrition fact label mandated the inclusion of the amount of potassium in packaged foods in the nutrition label [43].

#### Regulations and Standards

Regulations and standards provide reference for the production, packaging, trading, and use of salt substitutes and are issued by government departments to ensure safety and effectiveness. Regulations are mandatory and have legally binding effects. A total of 4 countries (the United States, Canada, China, and India; Table 1) have established regulations or standards for salt substitutes or have addressed salt substitutes in other regulations or standards.

First, the United States [44], India [45], and China [36] had clear but varied regulations on the composition of salt substitutes. For example, the commercial item description of salt substitutes developed by the US Department of Agriculture in 1997 divided salt substitutes into 2 categories, potassium chloride (type I) and potassium chloride with L-lysine (type II) [44]. China has established an industry standard (QB/*t* 2019-2020) on *low-sodium salt*, which stated that sodium chloride and potassium chloride should reach 65.0-80.0 g/100 g and 20.0-35.0 g/100 g on a dry basis, respectively [36]. In addition to active ingredients, these countries also imposed requirements on other ingredients, such as ingredients used for food grade bulking, anticaking agents, flavor enhancers, nutrition enhancers, and pollutants. For example, India not only requires salt substitutes to contain no more than 120 mg/100 g of sodium but also restricts the component of cations in some acidity regulators [45]. Canada has not developed standards for salt substitutes, but the *Labelling Requirements for Salt* issued by the Canadian Food Inspection Agency in 2019 stated that salt substitutes were generally sodium-reduced or sodium-free alternatives that usually contain potassium chloride. When a salt substitute was used as an ingredient in another food, it was an additive [46]. In addition, Goods and Services Identification Number of Salt Substitute released by the Canadian Food Inspection Agency classified salt substitutes as special dietary foods and food specialty preparations [47]. The regulations on salt substitute composition could be clearly seen in Table 1, whereas the regulations and standards on labeling are introduced in the next section.

**Table 1:** Regulations and standards for salt substitutes in countries.

Category and country			Title		Year		Department		Regulation and standard for salt substitutes
**Regulations and standards of formula**									
	United States	Commercial Item Description—Salt Substitutes [44]		1997		The US Department of Agriculture		Classification: the salt substitutes shall conform to the following list which shall be specified in the solicitation, contract, or purchase order: Type I: potassium chloride Type II: potassium chloride with L-lysine All ingredients, including food grade bulking and anticaking agents, used in the preparation of the salt substitutes shall be of Food Chemicals Codex purity or US Pharmacopeia-National Formulary quality. The active ingredient in the salt substitutes shall be potassium chloride. Type II salt substitutes shall include L-lysine. The salt substitutes may contain flavor enhancers and anticaking agents such as, but not limited to monopotassium glutamate, glutamic acid hydrochloride, tricalcium phosphate, and calcium stearate.	
	China	Light Industry Standard of the People’s Republic of China QB/*t* 2019-2020 Low Sodium Salt [36]		2020		Ministry of Industry and Information Technology of the People’s Republic of China		This standard specifies the sensory, physical, and chemical properties of; food additives used in; nutritional fortification of; and contaminants of salt substitutes. The dry basis of sodium chloride and potassium chloride should reach 65.0-80.0 g/100 g and 20.0-35.0 g/100 g, respectively.	
	India	Ministry of Health and Family Welfare (Food Safety and Standards Authority of India) Notification F. No. Stds/03/Notification (LS)/ FSSAI-2017 [45]		2017		Ministry of Health and Family Welfare (Food Safety and Standards Authority of India)		Salt substitutes may contain:(a) Colloidal silica or calcium silicate: not more than 1% m/m of the salt substitute mixture, individually or in combination.(b) Diluents: safe and suitable nutritive foods as normally consumed namely, sugars, cereal flour. The addition of iodine-containing compounds to salt substitutes shall be as per the Foods Safety and Standards Regulations, 2011. The sodium content of salt substitutes shall be not more than 120 mg/100 g of the salt substitute mixture. The composition of salt substitutes shall be as follows: Potassium sulphate, potassium, calcium or ammonium salts of adipic; glutamic; carbonic; succinic; lactic; tartaric; citric; acetic; or hydro-, chloric, or orthophosphoric acids, and/or good manufacturing practice, except that phosphorus not to exceed 4% m/m and NH4+ 3% m/m of the salt substitute mixture Magnesium salts of adipic; glutamic; carbonic; citric; succinic; acetic; tartaric; lactic; or hydro-, chloric, or orthophosphoric acids, mixed with other Mg-free salt substitutes as listed in 6.(1) (a), 6.(1) (c) and 6.(1) (d), and/or Mg++ to be not more than 20% m/m of the total of the cations K+, Ca++and NH4+ present in the salt substitute mixture and phosphorus not to exceed 4% m/m of the salt substitute mixture Choline salts of acetic, carbonic, lactic, tartaric, citric, or hydrochloric acids, mixed with other choline-free salt substitutes as listed in 6.(1) (a), 6.(1) (b) and 6.(1) (d), and/or the choline content not to exceed 3% m/m of the salt substitute mixture Free adipic, glutamic, citric, lactic, or malic acids—good manufacturing practice	
**Regulations and standards of labeling**									
	Canada	Labelling Requirements for Salt [46]		2019		Canadian Food Inspection Agency		Salt substitutes do not have a prescribed standard and are generally a sodium reduced or sodium free alternative. Salt substitutes usually contain potassium chloride. Table salt substitutes, while not required to, are permitted to contain added iodine.	
**Regulations and standards of product classification**									
	Canada	Goods and Services Identification Number (GSIN)—Salt Substitute [47]		2018		Public Services and Procurement Canada		GSIN^a^ Category: Goods GSIN Group Description: Subsistence GSIN Class Description: Special Dietary Foods and Food Specialty Preparations	

^a^GSIN: Goods and Services Identification Number.

#### Labels

Labels refer to the list of nutritional composition and health claims printed on packages, front-of-package labels, etc. Regulations on the labels of salt substitute products and the labels of packaged foods with added salt substitutes are part of the national strategy for salt substitutes to guide consumers in choosing or not choosing relevant products and are one of the important policy tools. Canada [46], the United States [48], Singapore [65], China [49], and India [45] have all regulated salt substitute labels (Table 2).

For salt substitutes, Canada required all the ingredients in the salt substitute to be labeled [46]. In China, salt substitute product labels must mention the potassium content and clearly indicate that salt substitutes should be used with caution by people for whom high potassium intake is not suitable, such as those who work at high temperatures, those who engage in high-intensity physical labor, those with kidney dysfunction, and those with hypertension who are taking antihypertensive medication [36,49].

For packaged foods containing salt substitutes, India required the labels to carry information on the presence of salt substitutes and the amount of potassium in them [45]. Because potassium chloride was not well known to consumers, research suggested that consumers may erroneously associate it with other chemicals and avoid consumption [66]. To change the negative public perception of potassium chloride and improve the health of the US population, NuTek Food Science, supported by food companies as well as public health organizations such as the Center for Science in the Public Interest and World Action on Salt & Health, submitted a citizen’s petition proposing that the US Food and Drug Administration rename “potassium chloride” as “potassium salt” in food labels in 2016, and the proposal was passed after a public consultation in 2020 [48,67]. Thereafter, potassium chloride in packaged foods could be renamed to “potassium salt,” but this is only a recommendation, not a mandatory responsibility, and is at the discretion of the food company. Canadian health promotion organizations such as Heart and Stroke and Hypertension Canada followed the lead of the United States and proposed to Health Canada and the Food Inspection Agency in 2019 to allow the use of the alternate name of “potassium chloride” [68]. The current Canadian law necessitates that the ingredient be identified by its common name along with “salt substitute” in parentheses. An example of this would be “potassium chloride (salt substitute)” [46]. Meanwhile, Canada has developed health claims for sodium and potassium, which can be made when processed foods meet the Food and Drugs Regulations’ requirements, such as “a healthy diet containing foods high in potassium and low in sodium may reduce the risk of hypertension. [Name of the food] is a good source of potassium and is low in sodium.” [50]. Health Canada’s reforms to nutrition labeling has been considering the inclusion of potassium in the nutrition fact label [39].

In addition, Singapore’s Healthier Choice Symbol (HCS) on packaged foods indicated to consumers which foods are healthier and more suitable choices for them [51]. The HCS label was found on approximately 4000 different foods and included 3 types of salt substitutes: Pagoda Less Sodium Mineral Salt, PanSalt, and GoodSalt [65].

**Table 2:** The regulations for the labels of salt substitute products and packaged foods with added salt substitutes.

Country		The regulation for labels
**Salt substitute products**		
	Canada [46]	All ingredients and their components must appear in the list of ingredients of table salt substitutes. This includes the declaration of iodide if present.
	India [45]	A declaration on the label as “low sodium salt substitute” or “low sodium dietetic salt” A declaration on the label regarding the amount of cations, that is, sodium, potassium, calcium, magnesium, ammonium, and choline/100 g (m/m), in the salt substitute mixture
**Packaged foods added salt substitutes**		
	United States [48]	Exercise enforcement discretion for the declaration of “potassium salt” in the place of “potassium chloride” in the ingredient statement of food labels when potassium chloride is used as an ingredient in the food. Potassium and sodium are listed on the nutrition fact label on packaged foods and beverages.
	Canada [46]	Salt substitute must be declared by its common name in the list of ingredients. The term “salt substitute” on its own would not be acceptable. However, it would be acceptable to declare the additive’s function in brackets after the common name, for example, “potassium chloride (salt substitute).” A salt substitute that meets the compositional and labeling criteria for a free of sodium or salt claim or for a low in sodium or salt claim may be represented as a food for special dietary use, such as “for salt free diets” or “for salt reduced diets.”
	China [49]	“低钠盐的产品标签中应标示钾的含量，并应清晰标示： ‘高温作业者、重体力劳动强度者、肾功能障碍者及服用降压药物的高血压患者等不适宜高钾摄入的人群应慎用。’” Translation: the product label of low-sodium salt should indicate the potassium content and should clearly indicate that “it should be used with caution by people who are not suitable for high potassium intake, such as those who work in high temperature, those who work with heavy physical strength, those who have kidney dysfunction and those who take antihypertensive drugs for hypertension.”

#### Food Reformulation, Cooperation With the Food Industry, and Media

Many companies in the food industry are trying to reduce the amount of sodium in processed foods through food reformulation, that is, by replacing some or the entire amount of salt with salt substitutes and finding formulations that maintain good organoleptic, microbiological, physical, and chemical properties and the safety and quality of food. Some reformulations were carried out by professional technicians or companies commissioned or supported by the government. The German Federal Ministry of Food and Agriculture produced healthy and safe sodium-reduced fish [52,53], semihard cheese [54,55], raw and salted meat products [56], etc.

In other countries such as Canada and Singapore, the government encourages food companies to use salt substitutes as a means of reducing salt in processed foods. In Singapore, in addition to the HCS labeling, the government is also running the Healthier Ingredient Promotion Scheme based on the HCS, which supports a variety of food companies, including salt producers, to innovate and market healthier and more locally flavored products with government funding that covers (1) marketing and publicity, (2) trade promotion, and (3) ingredient thematic promotion [57]. The salt selected for the program was IMI Lifestyle Products’ “GoodSalt.” The sodium content in GoodSalt was replaced by essential minerals such as potassium, magnesium, lysine, and iodine [69]. Finland is an example of countries that successfully promoted salt substitutes through the media, with a salt substitute called “Pansalt” being reported and promoted by mainstream media such as Helsingin Sanomat. With salt substitutes becoming well known in Finland, the media has significantly contributed to the realization of salt reduction in Finland [58].

### Salt Substitute Initiatives From 3 IGOs

Although IGOs are not countries, the initiatives proposed by them are often an important basis for regional or global food trade to ensure safety and health and are consciously abided by member states. These initiatives can be used as a basis for national legislation [24,70]. Therefore, this study also collected these initiatives on salt substitutes as a supplement to national initiatives (Multimedia Appendix 5 [59-62]).

Regarding the classification of salt substitutes, unlike Canada, the EU’s food additive standard stated that when table salt substitutes and minerals were used for flavor, mouthfeel, or nutritional purposes, they were considered substances and not food additives [59]. In addition, in terms of food reformation, the *Institut National de la Recherche Agronomique* commissioned by the EU produced sausages, in which 20% to 30% of sodium was replaced with potassium [60].

The CAC and the EAEU regulated the composition and labeling of salt substitutes and have similar requirements [61,62]. In terms of ingredients, they limited the maximum quantity of different substitutable ingredients; for example, they required “the choline content not to exceed 3% m/m of the salt substitute mixture.” For the labels of salt substitutes, the CAC required the mention of the name “low sodium salt substitute” or “low sodium dietetic salt,” whereas the EAEU required the mention of “the substitute of salt with low content of sodium” or “dietetic salt with low content of sodium.” Both regulations required companies to declare on the product label the full list of ingredients and the amount of cations in a 100 g mixture. For dietetic products containing salt substitutes, the presence of salt substitutes should be declared. The total potassium content and cation content in 100 g of the product should also be stated.

## Discussion

### Principal Findings

In this review, a total of 35 initiatives on salt substitutes from 11 countries and 3 IGOs were assessed in detail. Several major types of salt substitute initiatives, namely benefit-risk assessments and cautions; plans and actions; regulations and standards; labels; and food reformulation, cooperation with the food industry, and media, were summarized.

These initiatives had some significant features. First, the focus on salt substitutes reflects an interest in salt reduction. For most of the national initiatives that proposed salt substitutes as a means of salt reduction in the food industry, reducing the salt content of food has been regarded as a prerequisite. It has been shown that human preferences for salt taste could be shaped, with taste buds gradually adapting to saltier or less salty taste as dietary salt intake increases or decreases, respectively [71]. By contrast, replacing common salt with salt substitutes does not achieve the effects of lowering the taste threshold and changing the consumption pattern of the population. Salt substitutes as a means of salt reduction would only be considered in food categories if salt reduction is difficult because of microbial food safety, functional, and taste reasons.

Second, the mention of “potassium salt” or “potassium chloride (salt substitute)” as an alternative to “potassium chloride” on packaged food labels may be a new possible strategy. The International Food Information Council Foundation conducted a web-based survey of 1000 consumers and found that compared with potassium salt, consumers had a more negative association with potassium chloride, and their familiarity with “potassium” was very low [66]. However, there are still some issues to consider before implementation. For example, whether a name change would lead to a massive and rapid introduction of potassium salt products by the food industry, resulting in the lack of consideration of food safety and increased risk to susceptible groups, and the need to ensure appropriate mandatory nutrition labels, warning labels, and front-of-pack labels for potassium should be considered. Therefore, the implementation may need to be coupled with consumer education and monitoring.

Third, high-income countries are more concerned about the application and promotion of salt substitutes in the food industry, which is the industry where salt is mainly used. Several countries have funded research on innovative foods prepared with salt substitutes and cooperated with enterprises to encourage them to produce and sell packaged foods prepared with reduced salt and salt substitutes. However, some food companies have expressed the presence of barriers. For example, the effects of salt substitutes on product quality and taste have led to the frequent failure of companies’ research on reformulation with salt substitutes as well as increased costs. Companies have also expressed concerns about the consumer acceptability of salt substitutes [63]. It is important to consider the complex industry chain that includes salt substitute manufacturers, processed food manufacturers, retailers, consumers, media, and many others [72]. All stakeholders should work together to implement locally adapted initiatives to promote salt substitutes, such as establishing public-private partnerships and promoting salt substitutes through subsidies, tax incentives, awareness campaigns, promotions, and consumer education.

In middle-income countries, such as China, the source of sodium chloride intake is more likely to be household salt, especially in rural areas. A large randomized controlled trial in a Chinese population of >20,000 older people from 600 villages with a history of stroke or hypertension demonstrated that salt substitutes significantly reduced the risk of stroke, major cardiovascular events, and death (rate ratios=0.86, 0.87, and 0.88, respectively) [73]. Moreover, if all Chinese households were to switch to salt substitutes, 450,000 lives could be saved each year [10]. However, there are still some issues with the use of salt substitutes in households. For example, the possibility of adding more salt substitutes for taste demand; the bitter taste of potassium chloride, which may discourage some people with sensitive tastes from choosing it; the need to consider the balance between salt substitute intake and iodine intake in areas where iodine intake is inadequate; and a lack of recommendations or standards for the use of salt substitutes in the household preparation of some foods, such as cured meats, salted fish, and spicy cabbages, which may pose microbiological risks.

Fourth, there is a controversy among the benefit-risk assessments carried out in different countries with regard to the percentage of sodium expected to be replaced by potassium. In the United Kingdom, the assumption of replacing 15% to 25% of sodium with potassium gave an overall positive conclusion [28]. However, in Norway, assumptions of 30%, 50%, and 70% substitutions translated into potentially higher-risk conclusions in 2014 [31]. A new assessment in Norway in 2021 changed the assumption to 0% to 30% substitution of sodium with potassium [32]. Considering the different demographic profiles, sodium and potassium intakes of the population, average blood pressure, and epidemiology of cardiovascular disease in various countries, this study recommends the establishment of appropriate assumptions and benefit-risk assessments based on national realities.

In fact, benefit-risk assessments have always been focused on the possible harm of the use of potassium in salt substitutes. People with limitation of renal potassium excretion, such as patients with renal failure; those with diabetes mellitus; and those using potassium-sparing diuretics, angiotensin-converting enzyme inhibitors, or angiotensin receptor blockers, may experience cardiac arrhythmia and have an increased level of death risk after consuming excessive potassium [74]. Several cases of hyperkalemia caused by the use of salt substitutes have been reported in high-risk participants [75,76]. Nevertheless, 2 clinical trials from China conducted salt substitute interventions among older people in 600 villages and 48 residential older adult care facilities. No significant increase in adverse clinical outcomes was found in both studies, although the second study found that the use of salt substitutes may result in more frequent hyperkalemia biochemically [73,77]. These studies provided high level of evidence on the safety of salt substitute when it comes to promotion. Furthermore, monitoring of the use of salt substitutes after national or regional promotion campaigns is very important, namely population consumption of salt substitutes, levels of sodium and potassium consumed and excreted, changes in blood pressure, cardiovascular disease and mortality, and cases of adverse effects.

Fifth, media campaigns are an important part of consumer education for salt reduction efforts. For example, in Finland, where salt reduction efforts began early and achieved good results, there was a long-standing media campaign on salt reduction with a strong promotion of salt substitute brands [58,78]. By contrast, a company in India with a wide range of salt products, including salt substitutes, became the subject of fake news stating that their products contained toxic ingredients. Widely circulated on social media, the fake news caused panic among consumers, only to be clarified with a statement from the government [79].

Sixth, there are currently no proposed measures on the price control and taxation of salt substitutes in various countries. As of September 2020, the price of salt substitutes ranged from US $0.46 to US $87.00 per kg worldwide, which is higher than the price of regular salt [16]. In non–high-income countries, lowering the price of salt substitutes is undoubtedly an important factor in promoting salt substitutes. In high-income countries, the cost of input is an equally important consideration in whether companies choose to reformulate high-sodium foods with salt substitutes. If countries wish to actively promote the use of salt substitutes, they may consider controlling the price by reducing the tax. As of 2019, a total of 5 countries around the world tax high-sodium foods [7], yet there are currently no policies to increase or reduce taxes on salt substitutes in any individual country. At the same time, governments could invest resources to support technology development aimed at reducing the cost of producing salt substitutes.

So far, there have been few reports issued by governments on the effects of the implementation of salt substitute policies, such as changes in sodium and potassium intakes, cardiovascular health, and potential risks. Most of the salt substitute initiatives were proposed in recent years. It is possible that such assessments of effects may have not been published or remain internal government documents. By contrast, governments usually reported the overall effect of salt reduction, including changes in salt intake and the salt content of foods [7]. Nevertheless, the process and results of policy development in one country have implications for other countries, thanks to the extensive communication among stakeholders. According to press reports, after food and health organizations in the United States submitted a petition to the Food and Drug Administration to use an alternate name of potassium chloride, stakeholders in Canada also took action. Eventually, they both contributed to the renaming of potassium chloride [68]. The benefit-risk assessment published in the United Kingdom also had a wide impact, being mentioned in published documents in both Norway [32] and Ireland [30].

On the basis of the classification of country regions by the World Bank and WHO, salt substitutes are of interest in high-income countries and European countries or organizations [64,80] (Multimedia Appendix 4). Despite the neutral, positive, or conservative attitudes toward salt substitutes shown in the published initiatives in different countries, the publication itself represents a concern for salt substitutes. With higher importance attached to salt reduction and health, salt substitutes will be circulated and popularized around the world. Therefore, we call on more countries to implement salt substitute initiatives. It is necessary for all countries to conduct benefit-risk assessments and develop regulations or standards for salt substitutes and their labeling according to the circumstances. In addition to this, depending on the source of dietary salt (homemade food or packaged foods), the inclusion of salt substitutes in national salt reduction initiatives, appropriate interventions, and consumer education are also important approaches.

This review has some strengths and limitations. We did not coordinate with country program leaders, global experts in salt substitutes, or regional WHO representatives. However, a literature search that covered the peer-reviewed and gray literature, Google search, and government website search were used to obtain as much information as possible from the web. Owing to the limitations and time lags in the information published on the web, it is possible that not all country-level salt substitute initiatives are properly included. In addition, instead of multilingual translators, Google Translate was used to identify and filter non-English information, which might have resulted in the omission and misinterpretation of relevant initiatives in non–English-speaking countries. Furthermore, this review focused on official national initiatives on salt substitutes and did not include stakeholders, academic institutions, or subnational recommendations and initiatives, such as the salt substitute promotion initiative of the China National Salt Group in Beijing [81]. These subnational initiatives also have high relevance to the application and promotion of salt substitutes in the world and in the corresponding region and are references for many salt substitute initiatives at the national level.

### Conclusions

This scoping review has shown that although there are only a limited number of national initiatives focusing on salt substitutes at present, relevant initiatives are increasing year by year and involve multiple aspects, including regulations, guidelines, and cooperation with the industry. Given the great potential of salt substitutes in improving hypertension and stroke, we call on more countries to pay attention to and propose salt substitute initiatives. This requires the government to conduct a comprehensive evaluation and extensively cooperate with stakeholders, such as companies, media, and consumers.

## Appendix

Multimedia Appendix 1 PRISMA-ScR (Preferred Reporting Items for Systematic Reviews and Meta-Analyses Extension for Scoping Reviews) checklist.

Multimedia Appendix 2 Search strategies.

Multimedia Appendix 3 The complete salt substitute initiatives from nations and intergovernmental organizations.

Multimedia Appendix 4 Types of salt substitute initiatives.

Multimedia Appendix 5 The salt substitute initiatives by intergovernmental organizations.

## Abbreviations

CAC: Codex Alimentarius Commission
EAEU: Eurasian Economic Union
EU: European Union
HCS: Healthier Choice Symbol
IGO: intergovernmental organization
PRISMA-ScR: Preferred Reporting Items for Systematic Reviews and Meta-Analyses Extension for Scoping Reviews
UN: United Nations
WHO: World Health Organization

## Data Availability

All data generated or analyzed during this study are included in this published paper (and in Multimedia Appendices 1 to 5 ).
