# Supplementary material for: National Initiatives on Salt Substitutes: Scoping Review
Source: JMIR Public Health Surveill. 2023 Nov 17;9:e45266. doi: 10.2196/45266 (PMC10692885; doi:10.2196/45266)
Supplement: Multimedia Appendix 2 [file publichealth_v9i1e45266_app2.docx]

National Initiatives on Salt Substitutes: Scoping Review

Multimedia Appendix 2. Search strategies.

Part 1 (Page 1)

Concepts and keywords in government website search and Google search

Part 2 (Page 5)

Search steps in government website search

Part 3 (Page 6)

Search steps in Google search

Part 4 (Page 7)

Keywords in Pubmed, Web of Science and Google Scholar

Part 1

Concepts and keywords in government website search and Google search.

**Concepts**

salt substitutes, nations, intergovernmental organizations (IGOs), portal function, government portals, states, initiatives

**Keywords** of different aspects to the **concepts** and all the **nations** and **IGOs** involved in this study are as follows:

**1. salt substitutes**: “low-sodium salt”, “salt substitute”, “salt substitutes”, “salt substitution”, “potassium salt”, “mineral salt”, “sodium reduced salt”, “sodium potassium”

**2. nations**: “Afghanistan”, “Afghanistan”, “Albania”, “Algeria”, “Andorra”, “Angola”, “Antigua and Barbuda”, “Argentina”, “Armenia”, “Australia”, “Austria”, “Azerbaijan”, “Bahamas”, “Bahrain”, “Bangladesh”, “Barbados”, “Belarus”, “Belgium”, “Belize”, “Benin”, “Bhutan”, “Bolivia (Plurinational State of)”, “Bosnia and Herzegovina”, “Botswana”, “Brazil”, “Brunei Darussalam”, “Bulgaria”, “Burkina Faso”, “Burundi”, “Cabo Verde”, “Cambodia”, “Cameroon”, “Canada”, “Central African Republic”, “Chad”, “Chile”, “China”, “Colombia”, “Comoros”, “Congo”, “Costa Rica”, “Côte D'Ivoire”, “Croatia”, “Cuba”, “Cyprus”, “Czechia”, “Democratic People's Republic of Korea”, “Democratic Republic of the Congo”, “Denmark”, “Djibouti”, “Dominica”, “Dominican Republic”, “Ecuador”, “Egypt”, “El Salvador”, “Equatorial Guinea”, “Eritrea”, “Estonia”, “Eswatini”, “Ethiopia”, “Fiji”, “Finland”, “France”, “Gabon”, “Gambia (Republic of The)”, “Georgia”, “Germany”, “Ghana”, “Greece”, “Grenada”, “Guatemala”, “Guinea”, “Guinea Bissau”, “Guyana”, “Haiti”, “Honduras”, “Hungary”, “Iceland”, “India”, “Indonesia”, “Iran (Islamic Republic of)”, “Iraq”, “Ireland”, “Israel”, “Italy”, “Jamaica”, “Japan”, “Jordan”, “Kazakhstan”, “Kenya”, “Kiribati”, “Kuwait”, “Kyrgyzstan”, “Lao People’s Democratic Republic”, “Latvia”, “Lebanon”, “Lesotho”, “Liberia”, “Libya”, “Liechtenstein”, “Lithuania”, “Luxembourg”, “Madagascar”, “Malawi”, “Malaysia”, “Maldives”, “Mali”, “Malta”, “Marshall Islands”, “Mauritania”, “Mauritius”, “Mexico”, “Micronesia (Federated States of)”, “Monaco”, “Mongolia”, “Montenegro”, “Morocco”, “Mozambique”, “Myanmar”, “Namibia”, “Nauru”, “Nepal”, “Netherlands”, “New Zealand”, “Nicaragua”, “Niger”, “Nigeria”, “North Macedonia”, “Norway”, “Oman”, “Pakistan”, “Palau”, “Panama”, “Papua New Guinea”, “Paraguay”, “Peru”, “Philippines”, “Poland”, “Portugal”, “Qatar”, “Republic of Korea”, “Republic of Moldova”, “Romania”, “Russian Federation”, “Rwanda”, “Saint Kitts and Nevis”, “Saint Lucia”, “Saint Vincent and the Grenadines”, “Samoa”, “San Marino”, “Sao Tome and Principe”, “Saudi Arabia”, “Senegal”, “Serbia”, “Seychelles”, “Sierra Leone”, “Singapore”, “Slovakia”, “Slovenia”, “Solomon Islands”, “Somalia”, “South Africa”, “South Sudan”, “Spain”, “Sri Lanka”, “Sudan”, “Suriname”, “Sweden”, “Switzerland”, “Syrian Arab Republic”, “Tajikistan”, “Thailand”, “Timor-Leste”, “Togo”, “Tonga”, “Trinidad and Tobago”, “Tunisia”, “Türkiye”, “Turkmenistan”, “Tuvalu”, “Uganda”, “Ukraine”, “United Arab Emirates”, “United Kingdom of Great Britain and Northern Ireland”, “United Republic of Tanzania”, “United States of America”, “Uruguay”, “Uzbekistan”, “Vanuatu”, “Venezuela, Bolivarian Republic of”, “Viet Nam”, “Yemen”, “Zambia”, “Zimbabwe”.

**3. IGOs**: "Food and Agriculture Organization of the United Nations", "World Health Organization", "World Food Programme", "CAB International", "International Food Policy Research Institute", "European Union", "Council of Europe", "Central European Initiative", "European Free Trade Association", "Council of the Baltic Sea States", "Benelux", "Belgium–Luxembourg Economic Union", "British–Irish Council", "Nordic Council", "Northern Dimension Partnership in Public Health and Social Well-being", "Agency for International Trade Information and Cooperation", "Visegrád Group", "West Nordic Council", "Three Seas Initiative", "Asia Cooperation Dialogue", "Asia-Pacific Economic Cooperation", "Association of Southeast Asian Nations", "Bay of Bengal Initiative for Multi-Sectoral Technical and Economic Cooperation", "Trilateral Cooperation Secretariat", "South Asian Association for Regional Cooperation", "Gulf Cooperation Council", "Asia-Europe Foundation", "Central Asian Cooperation Organization", "Conference on Interaction and Confidence Building Measures in Asia", "Economic Cooperation Organization", "Eurasian Economic Union", "GUAM Organization for Democracy and Economic Development", "Organization of the Black Sea Economic Cooperation", "Organization of Turkic States", "Shanghai Cooperation Organisation", "TRACECA", "Union State", "North Atlantic Treaty Organization", "Organization for Security and Co-operation in Europe", "Union for the Mediterranean", "Indian Ocean Rim Association for Regional Cooperation", "Indian Ocean Commission", "Arctic Council", "Colombo Plan", "Pacific Islands Forum", "Secretariat of the Pacific Community", "Technical Centre for Agricultural and Rural Cooperation ACP-EU", "African Union", "Economic Community of West African States", "Conseil de l'Entente", "East African Community", "West African Economic and Monetary Union", "Southern African Development Community", "Intergovernmental Authority on Development", "International Conference on the Great Lakes Region", "Organization of American States", "Mercosur", "Andean Community of Nations", "Caribbean Community (CARICOM)", "Association of Caribbean States", "Organisation of Eastern Caribbean States", "Central American Parliament", "Bolivarian Alliance for the Americas", "Central American Integration System", "Community of Latin American and Caribbean States", "Pacific Alliance".

**4. portal function**: “health”, “public health”, “disease”, “medicine”, “food”, “drug”, “agriculture” “information”

**5. government portals**: “agency”, “ministry”, “administration”, “center”, “office”, “department”, “institute”, “organization”, “government”, “authority”

**6.** **states**: “state”, “federal”, “nation”, “national”

**7.** **initiatives**: “standard,” “regulation,” “requirement”, “plan”, “guideline”, “formula”, “composition”, “ingredient”, “assessment”, “caution”, “action”, “label”, “reformulation”, “cooperation”

Part 2

Search steps in government website search

The search of relevant data on 193 UN member states was conducted in 2 ways. First, all UN member states were showed in the keywords related to the concept “nation”. We found these nations’ government websites by searching nation AND “government portal” with Boolean operator. Then we searched for keywords related to "salt substitute" in the websites.

2. We found national government-related subsidiary organizations on Google search by searching keywords related to the follow concepts with Boolean operators:

nations AND portal function AND government portals

or

nations AND portal function AND government portals AND states

Then we searched keywords related to "salt substitutes" in the website

The search of relevant data on IGOs was conducted in 1 way.

All IGOs involved in our study were showed in the keywords related to the concept “IGOs”. We search these IGOs’ official websites by searching IGOs AND “official website” with Boolean operator.

and searched keywords related to "salt substitutes" in the websites.

Part 3

Search steps in Google search.

The keywords related to the follow concepts was searched with Boolean operators

1. salt substitutes AND initiatives

2. salt substitutes AND nations AND initiatives

3. salt substitutes AND IGOs AND initiatives

Part 4

Keywords in Pubmed, Web of Science and Google Scholar

Keywords in Pubmed

1. salt reduc*[Title/Abstract]

2. salt substitut*[Title/Abstract]

3. salt replac*[Title/Abstract]

4. low sodium salt[Title/Abstract]

5. sodium free salt[Title/Abstract]

6. mineral salt[Title/Abstract]

7. sodium reduced salt[Title/Abstract]

8. potassium salt[Title/Abstract]

9. potassium rich salt[Title/Abstract]

10. sodium potassium[Title/Abstract]

11. potassium sodium salt[Title/Abstract]

12. (nation** AND ((salt AND reduc*) OR (salt AND substitut*) OR (salt AND replac*) OR "low sodium salt" OR "sodium free salt" OR "mineral salt" OR "sodium reduced salt" OR "potassium salt" OR "potassium rich salt" OR "sodium potassium" OR "potassium sodium salt"))[Title/Abstract]

**nation: Keywords of different aspects to the concept “nation” in Part 1.

Keywords in Web of Science

1. TI= (salt AND reduc*) OR AB= (salt AND reduc*)

2. TI= (salt AND substitut*) OR AB= (salt AND substitut*)

3. TI= (salt AND replac*) OR AB= (salt AND replac*)

4. TI= "low sodium salt" OR AB= "low sodium salt"

5. TI= "sodium free salt" OR AB= "sodium free salt"

6. TI= "mineral salt" OR AB= "mineral salt"

7. TI= "sodium reduced salt" OR AB= "sodium reduced salt"

8. TI= "potassium salt" OR AB= "potassium salt"

9. TI= "potassium rich salt" OR AB= "potassium rich salt"

10. TI= "sodium potassium" OR AB= "sodium potassium"

11. TI= "potassium sodium salt" OR AB= "potassium sodium salt"

12. TI= (nation** AND ((salt AND reduc*) OR (salt AND substitut*) OR (salt AND replac*) OR "low sodium salt" OR "sodium free salt" OR "mineral salt" OR "sodium reduced salt" OR "potassium salt" OR "potassium rich salt" OR "sodium potassium" OR "potassium sodium salt")) OR AB= (nation** AND (salt AND reduc*) OR (salt AND substitut*) OR (salt AND replac*) OR "low sodium salt" OR "sodium free salt" OR "mineral salt" OR "sodium reduced salt" OR "potassium salt" OR "potassium rich salt" OR "sodium potassium" OR "potassium sodium salt"))

**nation: Keywords of different aspects to the concept “nation” in Part 1.

Keywords in Google Scholar

1. (intitle:(salt AND reduc*)) OR (abstract:(salt AND reduc*))

2. (intitle:(salt AND substitut*)) OR (abstract:(salt AND substitut*))

3. (intitle:(salt AND replac*)) OR (abstract:(salt AND replac*))

4. (intitle:("low sodium salt")) OR (abstract:("low sodium salt"))

5. (intitle:("sodium free salt")) OR (abstract:("sodium free salt"))

6. (intitle:("mineral salt")) OR (abstract:("mineral salt"))

7. (intitle:("sodium reduced salt")) OR (abstract:("sodium reduced salt"))

8. (intitle:("potassium salt")) OR (abstract:("potassium salt"))

9. (intitle:("potassium rich salt")) OR (abstract:("potassium rich salt"))

10. (intitle:("sodium potassium")) OR (abstract:("sodium potassium"))

11. (intitle:("potassium sodium salt") OR (abstract:("potassium sodium salt"))

12. intitle:(nation** AND ((salt AND reduc*) OR (salt AND substitut*) OR (salt AND replac*) OR "low sodium salt" OR "sodium free salt" OR "mineral salt" OR "sodium reduced salt" OR "potassium salt" OR "potassium rich salt" OR "sodium potassium" OR "potassium sodium salt"))

13. abstract:(nation** AND ((salt AND reduc*) OR (salt AND substitut*) OR (salt AND replac*) OR "low sodium salt" OR "sodium free salt" OR "mineral salt" OR "sodium reduced salt" OR "potassium salt" OR "potassium rich salt" OR "sodium potassium" OR "potassium sodium salt"))

**nation: Keywords of different aspects to the concept “nation” in Part 1.
