# Supplementary material for: National Initiatives on Salt Substitutes: Scoping Review
Source: JMIR Public Health Surveill. 2023 Nov 17;9:e45266. doi: 10.2196/45266 (PMC10692885; doi:10.2196/45266)
Supplement: Multimedia Appendix 3 [file publichealth_v9i1e45266_app3.docx]

National Initiatives on Salt Substitutes: Scoping Review

Multimedia Appendix 3. The complete salt substitute initiatives from nations and intergovernmental organizations.

| Countries or intergovernmental organizations | Characteristics of national salt substitute initiatives | Title | Year | Department | Initiatives | | | |
| --- | --- | --- | --- | --- | --- | --- | --- | --- |
| *Countries* | | | | | | | | |
| United Kingdom | Risk-benefit assessments | Potassium-based sodium replacers: assessment of the health benefits and risks of using potassium-based sodium replacers in foods in the UK [28]  URL: https://assets.publishing.service.gov.uk/government/uploads/system/uploads/attachment_data/file/660526/SACN_COT_-_Potassium-based_sodium_replacers.pdf [accessed Mar 22, 2022] | 2017 | The Scientific Advisory Committee on Nutrition (SACN) and the Committee on Toxicity of Chemicals in Food, Consumer Products and the Environment (COT) | SACN and COT used the Benefit-Risk Analysis for Foods (BRAFO)1 methodology to evaluate the benefits and risks of substitution of 15 to 25% of sodium by potassium in UK General population.  Conclusion At a population level, the potential benefits of using potassium-based sodium replacers to help reduce sodium in foods outweigh the potential risks. The beneficial effects at an individual level are likely to be small in size but will impact a large proportion of the population. The government should consider encouraging food companies to explore the use of potassium-based sodium replacers to help reduce sodium levels in foods. Risk managers should consider how to monitor the level of substitution of potassium for sodium in foods and the types of foods in which substitution is used. If the age structure of the UK population, the percentage of people with chronic kidney disease (currently approximately 1% of the UK population), or potassium intakes become materially different from those assumed for the modelling performed for this benefit-risk assessment, the government should reassess the balance between benefits and risks. | | | |
|  | Plans and actions, cooperation with the food industry | Salt reduction targets for 2024 [38]  URL: https://www.gov.uk/government/publications/salt-reduction-targets-for-2024 [accessed Mar 20, 2022] | 2020 | Public Health England | PHE’s preferred approach is for businesses to gradually reduce the overall saltiness of their products allowing for people’s palates to adjust to less salty foods. It is a business decision if and how they wish to use sodium replacers. | | | |
|  | Cooperation with the food industry | Summary of development of 2024 salt reduction targets [63]  URL: https://assets.publishing.service.gov.uk/government/uploads/system/uploads/attachment_data/file/915402/2024_salt_reduction_targets_Appendix-1_070920-FINAL.pdf [accessed Mar 20, 2022] | 2020 | Public Health England | Use of sodium replacers Industry feedback suggested that potassium-based or other sodium replacers were currently used in a limited capacity (for example in pelleted snacks and soup). A number of businesses commented that trials of the use of sodium replacers had been generally unsuccessful due to the impact on product quality and taste/flavour (for example in cheese, cakes and biscuits). Increased cost was also mentioned as a barrier. Some businesses were continuing to explore the use of sodium replacers (for example as a raising agent in bread). Consumer acceptability concerns were cited as a particular barrier, relating to the taste of products (the use of sodium replacers can result in a metallic taste), and incompatibility with a ‘clean label’. Suppliers of sodium replacers highlighted the acceptable use of their products in categories such as bread, meat and cheese, and indicated that work with businesses to explore the use of sodium replacers was ongoing. Health NGOs considered that for food categories where salt reduction still proves to be challenging, replacing their salt content with potassium-based or other sodium replacers should be encouraged as a short-term measure, and where sodium replacers are an appropriate substitute, their use should be promoted. | | | |
| Germany | Food reformulation | The National Reduction and Innovation Strategy: Less sugar, fats and salt in processed foods  URL: https://www.bmel.de/EN/topics/food-and-nutrition/healthy-diet/reduction-innovation-strategy-less-sugar-fat-salt.html#:~:text=The%20aim%20is%20that%20processed%20foodstuffs%20contain%20less,focus%20on%20products%20targeted%20at%20children%20and%20adolescents. [accessed Apr 20, 2022] | 2021 | Federal Ministry of Food and Agriculture（BMEL） | Since 2016, the BMEL has funded innovative research related to the reduction of sugar, fats and salt. Three of them are related to salt substitution. Strategies for salt reduction in meat products (17.10.2016 - 30.11.2019) [56] Salt reduction in selected fish products by means of salt substitutes (01.10.2016 - 31.03.2018) [52, 53] Strategies for salt reduction in semi-hard cheese (01.07.2016 - 30.06.2018) [54, 55] | | | |
|  |  |  |  |  | Further research funded by the BMEL has been/is currently addressing this topic:  Sensory optimisation of sodium-reduced semi-hard cheese (2021-2023) | | | |
| Norway | Risk-benefit assessments | Risk- benefit assessment of potassium chloride as replacement for sodium chloride [31]  URL: https://vkm.no/english/riskassessments/allpublications/riskbenefitassessmentofpotassiumchlorideasreplacementforsodiumchloride.4.27ef9ca915e07938c3b29bc0.html [accessed Apr 30, 2022] | 2014 | The Norwegian Food Safety Authority and The Norwegian Directorate of Health requested Norwegian Scientific Committeefor Food and Environment (VKM) | The assessment covers possible effects on food safety by full or partial replacement of sodium chloride (NaCl) by potassium chloride (KCl). VKM concluded that it is reasonable to anticipate that the percentage of persons likely to face an increased risk is far greater than the percentage of persons likely to benefit from this measure. | | | |
|  | Risk-benefit assessments | Potassium chloride as substitute for salt - a benefit-risk assessment [32]  URL: https://vkm.no/english/riskassessments/allpublications/potassiumchlorideassubstituteforsaltabenefitriskassessment.4.20bfba7e17a31ff03a53dd7.html [accessed Apr 30, 2022] | 2021 | The Norwegian Food Safety Authority and The Norwegian Directorate of Health requested Norwegian Scientific Committeefor Food and Environment | On behalf of the Norwegian Directorate of Health, VKM shall assess the benefits and risks of replacing salt (sodium chloride) with potassium chloride in food and drink. | | | |
| Ireland | Risk-benefit assessments | Salt and Health: Review of the Scientific Evidence and Recommendations for Public Policy in Ireland [29]  URL: https://www.fsai.ie/uploadedFiles/Science_and_Health/salt_report-1.pdf [accessed Mar 22, 2022] | 2005 | Food Safety Authority of Ireland | The Scientific Committee of Ireland discussed a wide range of potential initiatives that might be considered in developing measures to reduce population salt intake. Among potential initiatives discussed were the following: • Promotion of the use of low salt substitutes and herbs/spices. Regarding the use of low sodium salt, the Committee was of the opinion that this approach to reducing population dietary sodium cannot be endorsed at this time. Concerns were raised about the possible vulnerability of certain population sub-groups (including those with Type 1 diabetes, chronic renal insufficiency, end stage renal disease, severe heart failure and adrenal insufficiency) to high potassium load from these salt substitutes. It was also noted that the use of salt substitutes does not address the need to reduce salt taste thresholds in the population. | | | |
|  | Cooperation with the food industry | Salt Reduction Undertakings by the Food Industry – Update period August 2007-August 2008  URL: https://www.fsai.ie/science_and_health/salt_and_health/objectives_of_salt_programme.html [accessed Mar 22, 2022] | 2008 | Food Safety Authority of Ireland | To support Food Safety Authority of Ireland on salt reduction, Food service  supplier "Horgan’s Delicatessen Suppliers" undertaked to request that each supplier notifies any undertakings they had made in relation to reducing the use of salt in products produced, whether it be the introduction of low in salt product substitutes or the reduction of salt in current products where technically possible from 2005. And the achievements from 2006 to 2007 included a research which over 80% of suppliers responsed to. They found that where low in salt alternative products are not available, it has been noted that technically it would not be possible to produce the products with a lower level of salt without adversely affecting the product. | | | |
|  | Cooperation with the food industry | Salt Reduction Programme (SRP) – 2010 to 2011  URL: https://www.fsai.ie/science_and_health/salt_and_health/objectives_of_salt_programme.html [accessed Mar 22, 2022] | 2011 | Food Safety Authority of Ireland | The FSAI has advised the industry against the use of salt substitute’s for reduction of sodium in products. | | | |
|  | Risk-benefit assessments | Salt and Health: Review of the Scientific Evidence and Recommendations for Public Policy in Ireland (Revision 1) [30]  URL: https://www.fsai.ie/science_and_health/salt_and_health/the_science_of_salt_and_health.html [accessed Mar 22, 2022] | 2016 | Food Safety Authority of Ireland | Based on current scientific literature, the Scientific Committee concludes that there is scope for increased intake of potassium in the Irish diet through consumption of unprocessed or unrefined fruits, vegetables and nuts. The use of potassium-based salt replacement ingredients by the food industry could also help supplement intakes of potassium by the Irish population. However, potassium-based salt replacement ingredients should only be used by the food industry where reduction of sodium could be detrimental to food safety and/or the physical or organoleptic properties of foods. Furthermore, potassium-based salt replacement ingredients should not be used for the sole purpose of flavour maintenance, i.e. saltiness or flavour enhancement. There continues to be a requirement for the food industry to work on reducing the salt taste thresholds of the Irish population. The Scientific Committee recommends that the FSAI should: • Issue guidelines to the food industry on the use of potassium and other mineral-based salt replacement ingredients.These guidelines should consider the following: – Possible effects of the use of these ingredients on vulnerable groups – Types of replacement ingredients required by the food industry – Types of foods in which these ingredients would be used and at what levels – Likely reductions in salt levels in these foods – Impact on actual sodium reduction in foods – Impact on potassium intakes in the population • Guidelines to industry on the use of potassium and other mineral-based salt replacement ingredients should be made available to all vulnerable groups • Continue to monitor the sodium and potassium content of food and periodically re-evaluate intakes | | | |
| Finland | Cooperation with the food industry, media | Sodium Intake and Hypertension [58] | 2006 | University of Helsinki, University of Kuopio | Thanks to the extensive reports on the interventions in Finland of sodium-reduced, potassium-, and magnesiumenriched healthier salt alternatives, called ‘‘mineral salt’’ or ‘‘Pansalt,’’ Helsingin Sanomat has increased the interest of the population and governmental organizations in salt. With very few exceptions, smaller newspapers as well as TV and radio channels have more or less taken the same position as Helsingin Sanomat in the salt issues. Since January 1978, there have been hundreds of reports on both the harmful effects of salt and on the availability of healthier, goodtasting alternatives. | | | |
| United States | Regulations and standards | Commercial Item Description - salt substitutes [44]  URL: https://www.ams.usda.gov/sites/default/files/media/CID%20Salt%20Substitute.pdf [accessed May 25, 2022] | 1997 | The U.S. Department of Agriculture (USDA) | CLASSIFICATION. The salt substitutes shall conform to the following list which shall be specified in the solicitation, contract, or purchase order. Type I - Potassium chloride Type II - Potassium chloride with L-lysine All ingredients, including food grade bulking and anticaking agents, used in the preparation of the salt substitutes shall be of Food Chemicals Codex purity or U.S. Pharmacopeia-National Formulary quality. The active ingredient in the salt substitutes shall be potassium chloride. Type II salt substitutes shall include L-lysine. The salt substitutes may contain flavor enhancers and anticaking agents such as, but not limited to monopotassium glutamate, glutamic acid hydrochloride, tricalcium phosphate, and calcium stearate. | | | |
|  | Cautions | PART 184 - DIRECT FOOD SUBSTANCES AFFIRMED AS GENERALLY RECOGNIZED AS SAFE [37]  URL: https://www.ecfr.gov/current/title-21/chapter-I/subchapter-B/part-184 [accessed Apr 18, 2022] | 1983 | Code of Federal Regulations | The ingredient is used in food with no limitation other than current good manufacturing practice. The affirmation of potassium chloride as generally recognized as safe (GRAS) as a direct human food ingredient is based upon the following current good manufacturing practice conditions of use: The ingredient is used as flavor enhancer, flavoring agent, nutrient supplement, pH control agent and stabilizer or thickener. And the ingredient is used in food at levels not to exceed current good manufacturing practice. | | | |
|  | Labels, regulations and standards | The Use of an Alternate Name for Potassium Chloride in Food Labeling: Guidance for Industry [48]  URL: https://www.fda.gov/regulatory-information/search-fda-guidance-documents/guidance-industry-use-alternate-name-potassium-chloride-food-labeling [accessed Mar 22, 2022] | 2020 | U.S. Department of Health and Human Services, Food and Drug Administration and Center for Food Safety and Applied Nutrition | Exercise enforcement discretion for the declaration “potassium salt” in the place of “potassium chloride” in the ingredient statement of food labels when potassium chloride is used as an ingredient in the food. The important public health benefits for the U.S. population that could result from reduced sodium and increased potassium intake; the recognition that potassium chloride can substitute for sodium chloride in a variety of food manufacturing applications across a number of food categories; and the unlikelihood that the alternate name will mislead consumers. | | | |
|  | Plans and actions, labels | Dietary Guidelines for Americans, (2020-2025) [43]  URL: https://www.dietaryguidelines.gov/ [accessed Mar 22, 2022] | 2020 | U.S. Department of Agriculture and U.S. Department of Health and Human Services | Limit foods and beverages higher in sodium. Sodium—Less than 2,300 milligrams per day—and even less for children younger than age 14. Potassium is considered dietary components of public health concern for the general U.S. population because low intakes are associated with health concerns. Potassium and sodium are listed on the Nutrition Facts label on packaged foods and beverages | | | |
| Canada | Plans and actions, cooperation with the food industry | Sodium Reduction Strategy for Canada [39]  URL: https://www.canada.ca/en/health-canada/services/food-nutrition/healthy-eating/sodium/related-information/reduction-strategy/recommendations-sodium-working-group.html [accessed Apr 25, 2022] | 2010 | Health Canada | Food manufacturers should lead the implementation of this recommendation. In some cases, it will be necessary to find alternatives to salt or other sodium-containing ingredients due to microbial food safety or functionality reasons. However, it would be beneficial when formulating commercially prepared foods to first reduce the use of salt. Since it has been shown that, over time, the palate will condition itself to less salty tasting foods, salt alternatives should only be used when salt replacement is absolutely necessary. | | | |
|  | Labels |  |  |  | Implementation of this recommendation should be led by Health Canada. Reforms to nutrition labelling should also consider requiring the disclosure of the amounts of potassium, especially for products to which potassium salts have been added. The Food and Drug Regulations do not currently require that the Nutrition Facts table disclose the amount of potassium unless a claim is made about the amount of sodium in the food. This information is necessary to allow certain vulnerable populations to avoid high-potassium foods and for government officials to track changes in the amount of potassium in the food supply, particularly as many companies may start using more potassium-based salt substitutes. | | | |
|  | Cautions |  |  |  | Health Canada should take the lead in implementing this recommendation. It is recognized that the overall reduction of sodium in the food supply is desirable in order to achieve a meaningful reduction in the dietary sodium intake of Canadians. At the same time, it is not always possible simply to remove the salt or other sodium containing food additives because of the role they are playing in specific foods, particularly in relation to microbial food safety, food preservation, texture and flavour. In some of these cases, the salt and/or other sodium containing compounds that are removed must be replaced by other ingredients or food additives that will fulfill the same function. In order to ensure that this can occur in the food industry in a timely manner, certain regulatory changes may be needed to facilitate innovation. There is a need to streamline the current regulatory review process for food additives without compromising the rigour of the safety assessment, as well as to modernize the standards of identity for the use of ingredients to replace sodium, particularly when required for microbial food safety purposes. | | | |
|  | Food reformulation,  risk-benefit assessments |  |  |  | Research Recommendations: Alternatives to sodium, considering safety and functionality, including technological innovations and alternative food processing strategies Investigating the effectiveness, potential risks and benefits of non-sodium salts in food technology and food safety (e.g., potassium salts, multi-mineral salts, other flavour enhancers and flavour complements, etc.). | | | |
|  | Labels | Guidance for the Food Industry on Reducing Sodium in Processed Foods [50]  URL: https://www.canada.ca/en/health-canada/services/food-nutrition/legislation-guidelines/guidance-documents/guidance-food-industry-reducing-sodium-processed-foods-2012.html#a33 [accessed May 08, 2022] | 2012 | Health Canada | The purpose of this document is to provide guidance to the food industry for lowering the sodium levels in processed foods. Health Claims Related to Sodium The following statements are the permitted wording for the health claim for a low sodium diet and reduced risk of stroke and heart disease. In order to use one of these statements, the food must meet all of the relevant conditions set out for the claim. Please see actual text in the FDR for conditions to be met when making these health claims. (1) "A healthy diet containing foods high in potassium and low in sodium may reduce the risk of high blood pressure, a risk factor for stroke and heart disease. (Naming the food) is sodium-free." (2) "A healthy diet containing foods high in potassium and low in sodium may reduce the risk of high blood pressure, a risk factor for stroke and heart disease. (Naming the food) is low in sodium." (3) "A healthy diet containing foods high in potassium and low in sodium may reduce the risk of high blood pressure, a risk factor for stroke and heart disease. (Naming the food) is a good source of potassium and is sodium-free." (4) "A healthy diet containing foods high in potassium and low in sodium may reduce the risk of high blood pressure, a risk factor for stroke and heart disease. (Naming the food) is a good source of potassium and is low in sodium." (5) "A healthy diet containing foods high in potassium and low in sodium may reduce the risk of high blood pressure, a risk factor for stroke and heart disease. (Naming the food) is high in potassium and is sodium-free." (6) "A healthy diet containing foods high in potassium and low in sodium may reduce the risk of high blood pressure, a risk factor for stroke and heart disease. (Naming the food) is high in potassium and is low in sodium." | | | |
|  | Regulations and standards | GSIN (Goods and Services Identification Number) - Salt Substitute [47]  URL: https://buyandsell.gc.ca/procurement-data/goods-and-services-identification-number/gsin/N8940LA [accessed Apr 23, 2022] | 2018 | Public Services and Procurement Canada | GSIN Category Goods GSIN Group Description Subsistence  GSIN Class Description Special Dietary Foods and Food Specialty Preparations | | | |
|  | Regulations and standards | Labelling requirements for salt [46]  URL: https://inspection.canada.ca/food-labels/labelling/industry/salt/eng/1391790253201/1391795959629?chap=0 [accessed Apr 30, 2022] | 2019 | Canadian Food Inspection Agency | Salt substitutes do not have a prescribed standard and are generally a sodium reduced or sodium free alternative. Salt substitutes usually contain potassium chloride. Table salt substitutes, while not required to, are permitted to contain added iodine | | | |
|  | Labels |  |  |  | All ingredients and their components must appear in the list of ingredients of table salt substitutes. This includes the declaration of iodide if present. When a salt substitute is used as an ingredient in another food, it must be declared by its common name in the list of ingredients. The term "salt substitute" on its own would not be acceptable. However it would be acceptable to declare the additive's function in brackets after the common name, for example, "potassium chloride (salt substitute)". A salt substitute that meets the compositional and labelling criteria for a free of sodium or salt claim or for a low in sodium or salt claim may be represented as a food for special dietary use, such as "For Salt Free Diets" or "For Salt Reduced Diets" | | | |
|  | Plans and actions, cooperation with the food industry | Voluntary sodium reduction targets for processed foods 2020-2025  URL: https://www.canada.ca/en/health-canada/services/publications/food-nutrition/sodium-reduced-targets-2020-2025.html [accessed Apr 30, 2022] | 2020 | Health Canada | Health Canada acknowledges that reducing sodium can be challenging and thus encourages manufacturers explore different options to lower sodium levels in processed foods. In addition to simply use less salt, this could include using salt substitutes like potassium chloride where necessary. To help inform consumers of the role of potassium chloride in food, "salt substitute" can be listed in brackets following potassium chloride in the list of ingredients. | | | |
| Australia | Plans and actions, cautions | Healthy Food Partnership Reformulation Program: Implementation Plan [40]  URL: https://www.health.gov.au/resources/publications/partnership-reformulation-program-implementation-plan [accessed Apr 30, 2022] | 2020 | Australian Government Department of Health | In particular, if partial potassium-based replacement of sodium were to be implemented, it would be advisable to monitor for the increased consumption of potassium which may be a risk for at-risk populations. The Department has commenced work with the Australian Bureau of Statistics (ABS) to explore ways to monitor this potential risk. | | | |
|  | Risk-benefit assessments | Food Reformulation: Risk Assessment [33]  URL: https://www.health.gov.au/sites/default/files/documents/2021/03/partnership-reformulation-program-risk-assessment-report-food-reformulation-program-risk-assessment-report.pdf [accessed Apr 30, 2022] | 2020 | Australian Government Department of Health | Where nutrients such as sugar and sodium are reformulated with sugar and sodium substitutes, we question the impact of this, and the subsequent change to population consumption patterns. We don't eat single nutrients - it is important to consider the food as a whole - reducing public health sensitive nutrients does not necessarily mean that the resulting product is healthier or more nutritious overall. | | | |
|  | Cautions |  |  |  | Potassium-based replacement could, however, affect the health of people with major impairment of renal function because of Chronic Kidney Disease (CKD) or other morbidity, and those taking medications such as Angiotensin Converting Enzyme (ACE) inhibitors and potassium-sparing diuretics that reduce renal excretion of potassium. Most, but by no means all of these vulnerable individuals will be elderly. In theory, patients with diagnosed CKD and those taking medicines that predispose to hyperkalaemia could be advised by their doctors to avoid foods in which sodium has been replaced by potassium. However, this will only be practical if the food products concerned are clearly labelled, and suitable alternatives are readily available that do not contain potassium-based replacements. | | | |
|  |  |  |  |  | Monitor its application, along with temporal trends in the incidence of hyperkalaemia, both in patients with known vulnerability, and in those not previously recognised as being at risk | | | |
| Singapore | Cooperation with the food industry, media | Healthier Choice Symbol [51]  URL: https://www.hpb.gov.sg/food-beverage/healthier-choice-symbol [accessed Apr 13, 2022] | last update 31 March 2022 | Health Promotion Board | The Healthier Choice Symbol on packaged food products indicates that they are healthier options, and are an easy way for consumers to tell which food products are better for their diet than others! This empowers the individual to make informed food choices.Today, the Healthier Choice Symbol can be seen on about 4000 different food products, spanning across over 100 food categories such as convenience meals, sauces, beverages and breakfast cereals. Salt products included in are: 1. Pagoda LESS SODIUM MINERAL SALT； 2. PanSalt； 3. GoodSalt。 The Healthier Choice Symbol on packaged food products indicates that they are healthier options, and are an easy way for consumers to tell which food products are better for their diet than others! This empowers the individual to make informed food choices.Today, the Healthier Choice Symbol can be seen on about 4000 different food products, spanning across over 100 food categories such as convenience meals, sauces, beverages and breakfast cereals. Salt products included in are: | | | |
|  | Cooperation with the food industry, media | Healthier Ingredient Promotion Scheme [57]  URL: https://www.hpb.gov.sg/healthy-living/food-beverage/healthier-ingredient-schemes/about-the-healthier-ingredient-promotion-scheme [accessed Jul 05, 2022] | last updated on 31 Jan 2022 | Health Promotion Board | The Healthier Ingredient Development Scheme (HIDS) encourages suppliers and manufacturers to innovate and develop a wider variety of healthier ingredients and supports the industry in promoting the increased use of healthier ingredients by Singapore’s food service. The HIPS covers both oil and grain staples, mainly rice and noodles, as well as sugar-sweetened beverages, sauces, desserts, wholegrain products, sweet spreads and table salt. HIDS offers support under three categories: (1) Research, Product Development, Packaging and Certification, (2) Marketing and Publicity and (3) Trade Promotions (such as bulk purchase rebates and bonus incentives for incremental sales of healthier ingredient products).The healthier salt supported by HIDS is "Good Salt" produced by IMI Lifestyle Products，The product is described on IMI's official website as follows：GoodSalt is a low sodium iodized salt that tastes like regular salt. The sodium content in GoodSalt is replaced by essential minerals such as Potassium, Magnesium, Lysine and Iodine. | | | |
|  | Cautions | Medication Information Leaflet-Potassium Chloride [34]  URL: https://www.healthhub.sg/a-z/medications/368/Potassium-Chloride [accessed Apr 30, 2022] | 2019 | Pharmaceutical Society Of Singapore | What food or medication should I avoid when I take this medication? Do not use salt substitutes or consume low-sodium food products unless your doctor has advised you to do so, as these products may contain potassium. Check the labels carefully on all low-sodium food products before consuming. | | | |
|  | Cautions | Management of Chronic Heart Failure [35]  URL: https://www.moh.gov.sg/docs/librariesprovider4/guidelines/management-of-chronic-heart-failure---booklet.pdf [accessed Aug 05, 2022] | 2007 | Singapore Ministry of Health | Potassium supplementation is generally stopped after the initiation of Aldosterone antagonist (AA). Patients should be counselled to moderate consumption of foods high in potassium and salt substitutes. | | | |
| China | Plans and actions | China World Action on Salt and Health (CWASH) (2010-2020) [41]  URL: https://www.docin.com/p-977861860.html [accessed May 25, 2022] | 2010 | Chinese Center for Disease Control and Prevention, The George Institute for Global Health-China, Shanghai Academy of Ecological Health Sciences | 在中国有很大一部分人居住在农村地区，这些地区的情况和城市有很多不同。比如说，在中国很多农村地区，心血管疾病负担很重，而钠的摄入量非常高（约260mmol/24hrs），钾的摄入量又非常的低(约50mmol/24hrs)。在这些地区，由于几乎全部的食物都是在家里准备的，大部分的钠的摄入都来源于做饭时候放入的盐，因此从长远来看，很有可能可以通过普及低钠高钾的替代盐来达到控制饮食中钠和钾的摄入的目的，而这种方法的成本也是很低的。这种低钠高钾的替代盐在降低血压上的作用，曾经有一些专门报道，大范围的应用也有相当的前景。而且中国的食盐制造业是国家专营，之前在全国范围内食盐加碘运动的成功，也为这种全人群中调整食盐供给提供了先例，因此，在政府的支持下，想要达到全人群中食盐摄入量的降低相对可行而且需要的时间也会比较短。  Translation: To control the intake of sodium and potassium in the diet of residents in rural areas of China by popularizing salt substitutions with low sodium and high potassium. A large proportion of people in China live in rural areas, and the situation in these areas differs in many ways from urban areas. For example, in many rural areas of China, there is a high burden of cardiovascular disease, while sodium intake is very high (about 260 mmol/24hrs) and potassium intake is very low (about 50 mmol/24hrs). In these areas, since almost all food is prepared at home and most of the sodium intake comes from the salt put in the cooking, it is likely that in the long run, control of dietary sodium and potassium intake can be achieved by popularizing low sodium salt, which is also very low cost. The role of such low sodium salt in lowering blood pressure has been reported in some specific cases, and there is considerable promise for widespread application. Moreover, the salt manufacturing industry in China is a state franchise, and the success of previous nationwide salt iodization campaigns has provided precedents for such population-wide adjustments in salt supply, so it is relatively feasible and time-consuming to achieve population-wide reductions in salt intake with government support. | | | |
|  | Regulations and standards | Light Industry Standard of the People's Republic of China QB/T 2019-2020 Low sodium salt [36]  URL: https://www.cssn.net.cn/cssn/productDetail/30482f65c42915ff132748edc92a8009 [accessed May 10, 2022] | 2020 | Ministry of lndustry and Information Technology of the People's Republic of China | This standard specifies the low sodium salt, including sensory properties, physical and chemical properties, food additives and nutritional fortification (iodine fortification, potassium ferricyanide, sodium ferricyanide), contaminants (lead, total arsenic, cadmium, total mercury, barium), etc. The regulations of main components are 65.0 ~ 80.0g/100g sodium chloride on a dry basis and 20.0 ~ 35.0g/100g potassium chloride on a dry basis. | | | |
|  | Labels | National Standard of the People’s Republic of China GB 2721-2015 National Food Safety Standards Food Grade Salt [49]  URL: https://www.cssn.net.cn/cssn/productDetail/009c1bf59b4bbf0dc137ccab0fed67bc [accessed May 25, 2022] | 2015 | National Health and Family Planning Commission of the P.R.C. | 低钠盐的产品标签中应标示钾的含量，并应清晰标示：“高温作业者、重体力劳动强度着、肾功能障碍者及服用降压药物的高血压患者等不适宜高钾摄入的人群应慎用。“  Translation: The product label of low sodium salt should indicate the potassium content, and should clearly indicate that “it should be used with caution by people who are not suitable for high potassium intake, such as those who work in high temperature, those who work with heavy physical strength, those who have kidney dysfunction and those who take antihypertensive drugs for hypertension.” | | | |
| India | Regulations and standards | MINISTRY OF HEALTH AND FAMILY WELFARE (Food Safety and Standards Authority of India) NOTIFICATION F. No. Stds/03/Notification (LS)/ FSSAI-2017 [45]  URL: https://fssai.gov.in/upload/uploadfiles/files/Gazette_Notification_Special_Dietary_Food_22_06_2017.pdf [accessed May 20, 2022] | 2017 | MINISTRY OF HEALTH AND FAMILY WELFARE | Salt Substitutes—(1) The composition of salt substitutes shall be as follows: | | | |
|  |  |  |  |  | a | Potassium sulphate, potassium, calcium or ammonium salts of adipic, glutamic, carbonic, succinic, lactic, tartaric, citric, acetic, hydrochloric or ortho phosphoric acids, and/or | good manufacturing practice, except that Phosphorus not to exceed 4 per cent. m/m and NH_4_^+^ 3 per cent. m/m of the salt substitute mixture | |
|  |  |  |  |  | b | Magnesium salts of adipic, glutamic, carbonic, citric, succinic, acetic, tartaric, lactic, hydrochloric or orthophosphoric acids, mixed with other Mg-free salt substitutes as listed in (1)(a), (1)(c) and (1)(d), and/or | Mg^++^ to be not more than 20 per cent. m/m of the total of the cations K^+^, Ca^++^and NH_4_^+^ present in the salt substitute mixture and Phosphorus not to exceed 4 per cent. m/m of the salt substitute mixture | |
|  |  |  |  |  | c | Choline salts of acetic, carbonic, lactic, tartaric, citric or hydrochloric acids, mixed with other choline-free salt substitutes as listed in (1)(a), (1)(b) and (1)(d), and/or | The choline content not to exceed 3 per cent. m/m of the salt substitute mixture | |
|  |  |  |  |  | d | Free adipic, glutamic, citric, lactic or malic acids | good manufacturing practice | |
|  |  |  |  |  | (2) Salt substitutes may contain: (a) Colloidal silica or calcium silicate: not more than one per cent. m/m of the salt substitute mixture, individually or in combination. (b) Diluents: safe and suitable nutritive foods as normally consumed namely, sugars, cereal flour. (3) The addition of iodine-containing compounds to salt substitutes shall be as per the Foods Safety and Standards Regulations, 2011. (4) The sodium content of salt substitutes shall be not more than 120 mg/100 g of the salt substitute mixture. | | | |
|  |  |  |  |  | The addition of salt substitutes conforming to clause (6) of sub-regulation 2.9.30 of the Food Safety and Standards (Food Products Standards and Food Additives) Regulations, 2011 to a special dietary food with low sodium content is permitted and shall be limited by good manufacturing practice as provided under Food Safety and Standards Regulations, 2011. | | | |
|  | Labels |  |  |  | (5) Salt substitutes shall conform to the following specific provisions for the labelling in addition to the Food Safety and Standards (Packaging and Labelling) Regulations, 2011, namely:— (i) a declaration on the label as “low sodium salt substitute” or “low sodium dietetic salt"; (ii) a declaration on the label regarding the amount of cations (that is, sodium, potassium, calcium, magnesium, ammonium and choline/100 g (m/m) in the salt substitute mixture; | | | |
|  |  |  |  |  | The addition of the salt substitutes listed in clause (6) of sub-regulation 2.9.30 of the Food Safety and Standards (Food Products Standards and Food Additives) Regulations, 2011 shall be declared on the label. When a salt substitute, composed entirely or partially of a potassium salt, has been added, the total amount of potassium, expressed as mg cation per 100 g of the food as normally consumed, shall be declared on the label. | | | |
|  | Plans and actions, cautions | Do You Eat Right [42]  URL: https://fssai.gov.in/upload/knowledge_hub/852185f89a7fc009c5Book_Do_You_Eat_Right_16_10_2020.pdf [accessed May 25, 2022] | 2020 | the Food Safety and Standards Authority of India (FSSAI) | Low-sodium salt is a specially formulated salt that provides lower sodium than ordinary salt by partial replacement of sodium chloride with potassium, magnesium and calcium compounds. It is generally consumed by people with hypertension and high blood pressure. FSSAI is under the process of setting up standards for low sodium salts in India. Presently, there are few brands of low sodium salts available in India market. Advocation of low-sodium salts in which the sodium chloride (the harmful ingredient in salt) has been replaced with potassium chloride will help. However, it is advisable to consume it under medical supervision, and should be avoided by people with medical conditions like kidney problems, hyperkalemia etc. | | | |
| *Intergovernmental organizations* | | | | | | | | |
| Codex Alimentarius | Regulations and standards | Codex Alimentarius international food standards standard for special dietary foods with low-sodium content (including salt substitutes) CXS 53-1981 [61]  URL: https://tinyurl.com/2fmk3ntx [accessed May 20, 2022] | Adopted in 1981. Amended in 1983, 2019. | Food and Agriculture Organization of the United Nations, World Health Organization | The composition of salt substitutes shall be as follows:  Salt substitutes may contain:  (a) Colloidal silica or calcium silicate: not more than 1% m/m of the salt substitute mixture, individually or in combination.  (b) Diluents: safe and suitable nutritive foods as normally consumed (e.g. sugars, cereal flour).  The addition of iodine-containing compounds to salt substitutes shall be in conformity with the national legislation of the country where the product is sold.  The sodium content of salt substitutes shall be not more than 120 mg/100 g of the salt substitute mixture. | | | |
|  |  |  |  |  | (a) Potassium sulphate, potassium, calcium or ammonium salts of adipic, glutamic, carbonic, succinic, lactic, tartaric, citric, acetic, hydrochloric or orthophosphoric acids, and/or | | | Not limited, except that P not to exceed 4% m/m and NH+4 3% m/m of the salt substitute mixture |
|  |  |  |  |  | (b) Magnesium salts of adipic, glutamic, carbonic, citric, succinic, acetic, tartaric, lactic, hydrochloric or orthophosphoric acids, mixed with other Mg-free salt substitutes as listed in (a), (c) and (d), and/or | | | Mg^++^ to be not more than 20% m/m of the total of the cations K^+^, Ca^++^ and NH_4_^+^ present in the salt substitute mixture and P not to exceed 4% m/m of the salt substitute mixture |
|  |  |  |  |  | (c) Choline salts of acetic, carbonic, lactic, tartaric, citric or hydrochloric acids, mixed with other choline-free salt substitutes as listed in (a), (b) and (d), and/or | | | The choline content not to exceed 3% m/m of the salt substitute mixture |
|  |  |  |  |  | (d) Free adipic, glutamic, citric, lactic or malic acids | | | Not limited |
|  | Labels |  |  |  | The addition of the salt substitutes listed in paragraph 3.2 of this standard shall be declared on the label. When a salt substitute, composed entirely or partially of a potassium salt, has been added, the total amount of potassium, expressed as mg cation per 100 g of the food as normally consumed, shall be declared on the label. | | | |
|  |  |  |  |  | Salt Substitutes In addition to Sections 2, 3, 4.3 to 4.5 and 8 of the General Standard for the Labelling of Prepackaged Foods (CXS 1-1985) the following provisions shall apply: The name of the product shall be "low sodium salt substitute" or "low sodium dietetic salt". A complete list of ingredients shall be declared on the label. The amount of the cations (i.e. sodium, potassium, calcium, magnesium, ammonium and choline/100 g m/m in the salt substitute mixture shall also be declared on the label. | | | |
| European Union | Food reformulation | TERIFIQ [60]  URL: https://cordis.europa.eu/docs/results/289/289397/final1-final_report_terifiq_v1-0.pdf [accessed Apr 29, 2022] | 2016 | Institut National de la Recherche Agronomique (INRA) | It is possible to reduce total salt content in cooked sausages by 20% and 20 - 30% of sodium can be partially substituted by potassium. Thus, sodium and fat contents can be reduced by 40% and by 20%, respectively, when compared with the reference product without significantly affecting the sensory properties of cooked sausages. | | | |
|  | Regulations and standards | REGULATION (EC) No 1333/2008 OF THE EUROPEAN PARLIAMENT AND OF THE COUNCIL of 16 December 2008 on food additives [59]  URL: https://eur-lex.europa.eu/legal-content/EN/TXT/?uri=CELEX%3A32008R1333 [accessed Apr 30, 2022] | 2008 | Official Journal of the European Union | substances should not be considered as food additives when they are used for the purpose of imparting flavour and/or taste or for nutritional purposes, such as salt replacers, vitamins and minerals. | | | |
| Eurasian Economic Union | Regulations and standards | Technical regulation of the Customs Union on safety of specific types of specialized food products including the therapeutic and preventive dietary food [62]  URL: https://food.ec.europa.eu/system/files/2016-10/ia_eu-ru_sps-req_decision-34_annex_en.pdf [accessed Apr 28, 2022] | 2012 | the Council of the Eurasian Economic Commission | The composition of the salt substitutes must meet the requirements set in the following table of the present Technical Regulation.The content of sodium in salt substitutes must not exceed 120 mg/100 g of the mass of the salt substitute mixture. | | | |
|  |  |  |  |  | Composition of salt substitutes | | | |
|  |  |  |  |  | a) potassium sulfate, potassium, calcium and ammonium salts the adipic, glutamic and carbon, amber, lactic, wine, citric, acetous, hydrochloric, orthophosphoric acids | | | |
|  |  |  |  |  | b) magnesium salts of adipic, glutamic, carbon, citric, amber, acetous, wine, lactic, hydrochloric and orthophosphoric acids, in the mixture with other substitutes, which do not contain magnesium | | | |
|  |  |  |  |  | c) cholinic salts of acetous, carbon lactic, wine, citric and hydrochloric acids, in the mixture with other substitutes, which do not contain choline, | | | |
|  |  |  |  |  | d) free adipic, glutamic, citric, lactic and malonic acids | | | |
|  |  |  |  |  | The salt substitutes can also contain: 1) colloidal silicon or silicate of calcium not exceeding 1% of the mass of substitutes mixture, individually or in combination; 2) fillers: safe and suitable food products of common use (for example, sugar, grain flour). | | | |
|  |  |  |  |  | The addition of iodine containing compounds to the salt and the salt substitutes must meet the standards of the national laws of the Customs Union member state, in which the products are sold. | | | |
|  | Labels |  |  |  | In addition to all indications on the marking, which concern the dietetic product with low content of sodium (with exception of the salt substitutes as such) the following specific instructions concerning the marking must be fulfilled: 1) if substitutes are present, the information on the presence of salt substitutes listed in Appendix 2 to the present Technical Regulations must be indicated; 2) if salt substitute, which consists of potassium salt in full or in part has been added it is necessary to indicate on the marking the summary content of potassium expressed in milligrams of cation to 100 grams of products. The salt substitutes must be called “the substitute of salt with low content of sodium” or the “dietetic salt with low content of sodium”. The marking of salt substitutes must contain the full list of ingredients, and also the content of cations (sodium, potassium, calcium, magnesium, ammonium and choline) in 100 grams of the mass of the mixture of substitutes. | | | |
