# Supplementary figures and images for: National Initiatives on Salt Substitutes: Scoping Review
Source: JMIR Public Health Surveill. 2023 Nov 17;9:e45266. doi: 10.2196/45266 (PMC10692885; doi:10.2196/45266)

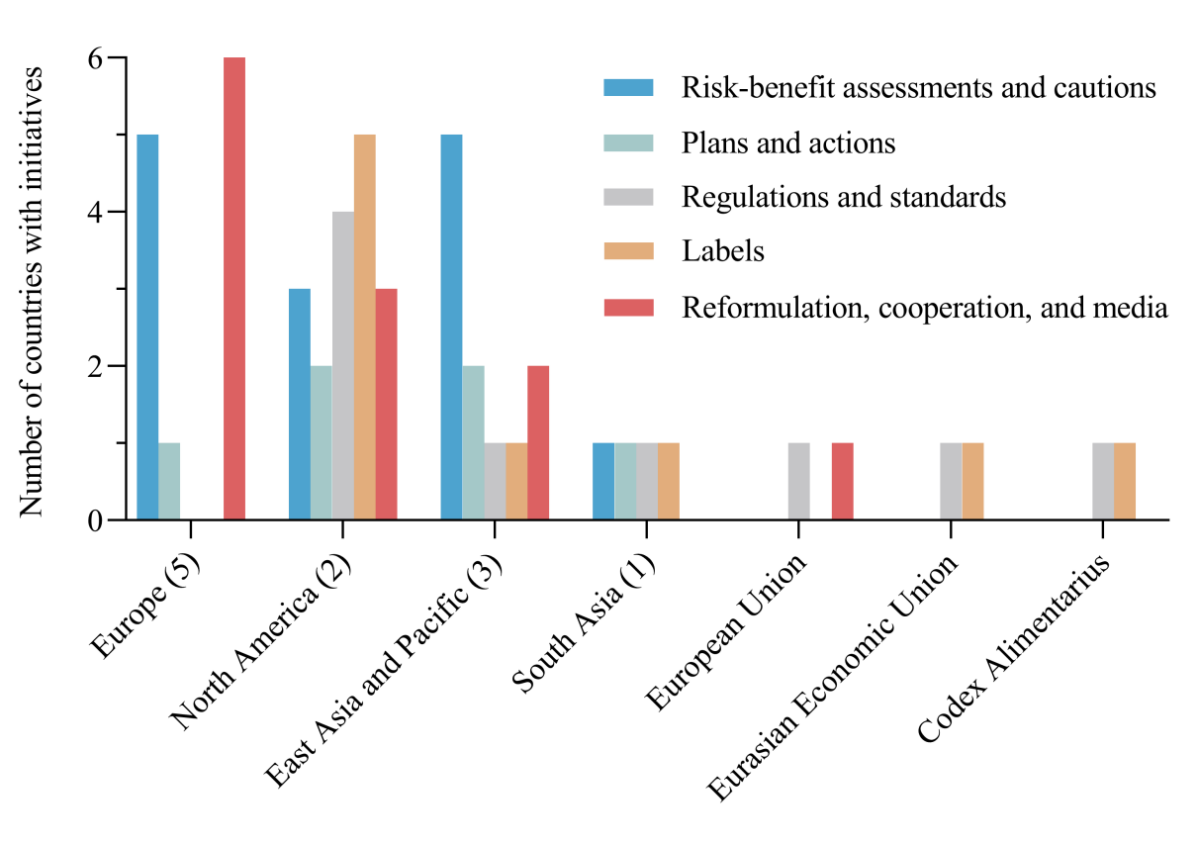

Supplement: Multimedia Appendix 4 [file publichealth_v9i1e45266_app4.png]
