# Supplementary material for: National Initiatives on Salt Substitutes: Scoping Review
Source: JMIR Public Health Surveill. 2023 Nov 17;9:e45266. doi: 10.2196/45266 (PMC10692885; doi:10.2196/45266)
Supplement: Multimedia Appendix 5 [file publichealth_v9i1e45266_app5.docx]

National Initiatives on Salt Substitutes: Scoping Review

Multimedia Appendix 4. The salt substitute initiatives in intergovernmental organizations.

| Intergovernmental organizations | Characteristics of salt substitute initiatives | Title | Year | Department | Initiatives | | |
| --- | --- | --- | --- | --- | --- | --- | --- |
| Codex Alimentarius | Regulations and standards | Codex Alimentarius international food standards standard for special dietary foods with low-sodium content (including salt substitutes) CXS 53-1981 [61]  URL: https://www.fao.org/fao-who-codexalimentarius/sh-proxy/en/?lnk=1&url=https%253A%252F%252Fworkspace.fao.org%252Fsites%252Fcodex%252FStandards%252FCXS%2B53-1981%252FCXS_053e.pdf [accessed May 20, 2022] | Adopted in 1981. Amended in 1983, 2019. | Food and Agriculture Organization of the United Nations, World Health Organization | The composition of salt substitutes shall be as follows:  Salt substitutes may contain:  (a) Colloidal silica or calcium silicate: not more than 1% m/m of the salt substitute mixture, individually or in combination.  (b) Diluents: safe and suitable nutritive foods as normally consumed (e.g. sugars, cereal flour).  The addition of iodine-containing compounds to salt substitutes shall be in conformity with the national legislation of the country where the product is sold.  The sodium content of salt substitutes shall be not more than 120 mg/100 g of the salt substitute mixture. | | |
|  |  |  |  |  | (a) Potassium sulphate, potassium, calcium or ammonium salts of adipic, glutamic, carbonic, succinic, lactic, tartaric, citric, acetic, hydrochloric or orthophosphoric acids, and/or | | Not limited, except that P not to exceed 4% m/m and NH+4 3% m/m of the salt substitute mixture |
|  |  |  |  |  | (b) Magnesium salts of adipic, glutamic, carbonic, citric, succinic, acetic, tartaric, lactic, hydrochloric or orthophosphoric acids, mixed with other Mg-free salt substitutes as listed in (a), (c) and (d), and/or | | Mg^++^ to be not more than 20% m/m of the total of the cations K^+^, Ca^++^ and NH_4_^+^ present in the salt substitute mixture and P not to exceed 4% m/m of the salt substitute mixture |
|  |  |  |  |  | (c) Choline salts of acetic, carbonic, lactic, tartaric, citric or hydrochloric acids, mixed with other choline-free salt substitutes as listed in (a), (b) and (d), and/or | | The choline content not to exceed 3% m/m of the salt substitute mixture |
|  |  |  |  |  | (d) Free adipic, glutamic, citric, lactic or malic acids | | Not limited |
|  | Labels |  |  |  | The addition of the salt substitutes listed in paragraph 3.2 of this standard shall be declared on the label. When a salt substitute, composed entirely or partially of a potassium salt, has been added, the total amount of potassium, expressed as mg cation per 100 g of the food as normally consumed, shall be declared on the label. | | |
|  |  |  |  |  | Salt Substitutes In addition to Sections 2, 3, 4.3 to 4.5 and 8 of the General Standard for the Labelling of Prepackaged Foods (CXS 1-1985) the following provisions shall apply: The name of the product shall be "low sodium salt substitute" or "low sodium dietetic salt". A complete list of ingredients shall be declared on the label. The amount of the cations (i.e. sodium, potassium, calcium, magnesium, ammonium and choline/100 g m/m in the salt substitute mixture shall also be declared on the label. | | |
| European Union | Food reformulation | TERIFIQ [60]  URL: https://cordis.europa.eu/docs/results/289/289397/final1-final_report_terifiq_v1-0.pdf [accessed Apr 29, 2022] | 2016 | Institut National de la Recherche Agronomique (INRA) | It is possible to reduce total salt content in cooked sausages by 20% and 20 - 30% of sodium can be partially substituted by potassium. Thus, sodium and fat contents can be reduced by 40% and by 20%, respectively, when compared with the reference product without significantly affecting the sensory properties of cooked sausages. | | |
|  | Regulations and standards | REGULATION (EC) No 1333/2008 OF THE EUROPEAN PARLIAMENT AND OF THE COUNCIL of 16 December 2008 on food additives [59]  URL: https://eur-lex.europa.eu/legal-content/EN/TXT/?uri=CELEX%3A32008R1333 [accessed Apr 30, 2022] | 2008 | Official Journal of the European Union | substances should not be considered as food additives when they are used for the purpose of imparting flavour and/or taste or for nutritional purposes, such as salt replacers, vitamins and minerals. | | |
| Eurasian Economic Union | Regulations and standards | Technical regulation of the Customs Union on safety of specific types of specialized food products including the therapeutic and preventive dietary food [62]  URL: https://food.ec.europa.eu/system/files/2016-10/ia_eu-ru_sps-req_decision-34_annex_en.pdf [accessed Apr 28, 2022] | 2012 | the Council of the Eurasian Economic Commission | The composition of the salt substitutes must meet the requirements set in the following table of the present Technical Regulation.The content of sodium in salt substitutes must not exceed 120 mg/100 g of the mass of the salt substitute mixture. | | |
|  |  |  |  |  | Composition of salt substitutes | Application conditions | |
|  |  |  |  |  | 1 | 2 | |
|  |  |  |  |  | a) potassium sulfate, potassium, calcium and ammonium salts the adipic, glutamic and carbon, amber, lactic, wine, citric, acetous, hydrochloric, orthophosphoric acids | Not limited, however the phosphorus content in the mixture of substitutes should not exceed 4%, and for NH4 + 3% of the total mass of substitutes. | |
|  |  |  |  |  | b) magnesium salts of adipic, glutamic, carbon, citric, amber, acetous, wine, lactic, hydrochloric and orthophosphoric acids, in the mixture with other substitutes, which do not contain magnesium | The content Mg must not exceed 20% in the mass of the general content of cations of K+, Ca, NH4 present in the mixture of substitutes, and the content of P must not exceed 4% of the total mass of substitutes. | |
|  |  |  |  |  | c) cholinic salts of acetous, carbon lactic, wine, citric and hydrochloric acids, in the mixture with other substitutes, which do not contain choline, | The content of choline must not exceed 3% of the mass of substitutes mixture | |
|  |  |  |  |  | d) free adipic, glutamic, citric, lactic and malonic acids | Not limited | |
|  |  |  |  |  | The salt substitutes can also contain: 1) colloidal silicon or silicate of calcium not exceeding 1% of the mass of substitutes mixture, individually or in combination; 2) fillers: safe and suitable food products of common use (for example, sugar, grain flour). | | |
|  |  |  |  |  | The addition of iodine containing compounds to the salt and the salt substitutes must meet the standards of the national laws of the Customs Union member state, in which the products are sold. | | |
|  | Labels |  |  |  | In addition to all indications on the marking, which concern the dietetic product with low content of sodium (with exception of the salt substitutes as such) the following specific instructions concerning the marking must be fulfilled: 1) if substitutes are present, the information on the presence of salt substitutes listed in Appendix 2 to the present Technical Regulations must be indicated; 2) if salt substitute, which consists of potassium salt in full or in part has been added it is necessary to indicate on the marking the summary content of potassium expressed in milligrams of cation to 100 grams of products. The salt substitutes must be called “the substitute of salt with low content of sodium” or the “dietetic salt with low content of sodium”. The marking of salt substitutes must contain the full list of ingredients, and also the content of cations (sodium, potassium, calcium, magnesium, ammonium and choline) in 100 grams of the mass of the mixture of substitutes. | | |
